# Middle Holocene plant cultivation on the Atlantic Forest coast of Brazil?

**DOI:** 10.1098/rsos.180432

**Published:** 2018-09-05

**Authors:** Luis Pezo-Lanfranco, Sabine Eggers, Cecilia Petronilho, Alice Toso, Dione da Rocha Bandeira, Matthew Von Tersch, Adriana M. P. dos Santos, Beatriz Ramos da Costa, Roberta Meyer, André Carlo Colonese

**Affiliations:** 1 Laboratório de Antropologia Biológica, Departamento de Genética e Biologia Evolutiva, Instituto de Biociências – Universidade de São Paulo, Rua do Matão 277, 05508-900, Cidade Universitária USP, São Paulo, Brazil; 2 Naturhistorisches Museum Wien, Anthropologische Abteilung, Burgring 7, 1010 Vienna, Austria; 3 BioArCh, Department of Archaeology, University of York, York YO10 5DD, UK; 4 Universidade da Região de Joinville, Mestrado em Patrimônio Cultural e Sociedade, Rua Paulo Malschitzki 10, Zona Industrial Norte, 89219-710, Joinville, Santa Catarina, Brazil; 5 Museu Arqueológico de Sambaqui de Joinville, Rua Dona Francisca 600, Centro, 89201-250, Joinville, Santa Catarina, Brazil

**Keywords:** early plant cultivation, South America, shellmounds, dental pathology, stable isotopes

## Abstract

This work provides robust oral pathology and stable isotope evidence on Bayesian mixing model for an unexpectedly high consumption of carbohydrates by a Middle Holocene coastal population of the Atlantic Forest of South America, an area traditionally viewed as peripheral to early centres of food production on the continent. A diversified economy with substantial consumption of plant resources was in place at the shellmound (or *sambaqui*) of Morro do Ouro, in Babitonga Bay, and supported a dense population at *ca* 4500 cal BP. This dietary composition is unique when compared with that of other contemporary and later groups in the region, including peoples who used ceramics and domesticated crops. The results corroborate independent dietary evidence, such as stone tool artefacts for plant processing and plant microremains in dental calculus of the same individuals, and suggest plant cultivation possibly took place in this region at the same time as the development of early agriculture in Amazonia and the La Plata Basin. Our study situates the Atlantic Forest coast of Brazil on the map of early plant management in the Neotropics.

## 1. Introduction

Food production fuelled population growth and the emergence of social complexity in pre-Columbian South America, from the Andes to lowland regions of the continent [1–3], leaving a longstanding legacy in regional biodiversity, cultural landscapes and traditional knowledge [4–8]. However, the nature, time and place of early plant cultivation and the development of independent centres of food production in the tropics are still matters of debate [3]. The narrow coastal strip of the Atlantic Forest, one of the world's most diverse tropical biomes [9], has supported human societies since the Middle Holocene, but their interaction with plant resources is still poorly understood [10,11]. As a result, this region has only been given cursory consideration, and it is viewed as peripheral to early centres of plant management and cultivation [3,12,13].

Shellmounds and middens, also known as *sambaquis* [14], are distinctive archaeological features of the Atlantic Forest formed by pre-ceramic coastal populations between *ca* 8000 and 1000 years ago [14,15], making them contemporaneous with the establishment of mixed economies and sedentary villages in the Andes and along major river basins of the continent [2,8,16]. The high frequency and large volume of some *sambaquis* on the southern coast of what is today Brazil, containing hundreds of human burials, have been taken as evidence for high population density, monumental architecture and social complexity during the Middle to Late Holocene [14,17]. These populations would have maintained economies founded primarily on the exploitation of rich marine ecosystems [18–20]. Increasing archaeobotanical studies, nevertheless, have provided a much greater appreciation of the dietary diversity among *sambaqui* builders. Remains of root crops, herbaceous plants and fruits with unknown domestication stages, consistent with yam (*Dioscorea* sp.), palms (Arecaceae), myrtle (Myrtaceae) and Annonaceae, have been found in several sites [10,11]. Similarly, starch grains and phytoliths compatible with maize (*Zea mays*), sweet potato (*Ipomoea batatas*), palms (Arecaceae), yam (*Dioscorea* sp.) and Araceae have been detected in dental calculus of Middle and Late Holocene *sambaqui* individuals along the southeastern coast of Brazil [21,22], who were often affected by relatively higher frequencies of oral pathologies [23]. Moreover, grinding stones and mortars presumably used for processing plant resources are commonly reported at *sambaqui* sites, indirectly reflecting investments in plant exploitation [11,17]. These independent lines of evidence indicate that plant resources were important components in *sambaqui* societies, and support the hypothesis that low-level food production [24] took place among these coastal groups.

We performed oral health and stable isotope analyses on human individuals from the *sambaquis* of Morro do Ouro (MO) and Rio Comprido (RC) in Babitonga Bay, southern Brazil, to unveil the dietary behaviour of human individuals during the Middle–Late Holocene. Our analyses revealed high levels of dental caries and evidence of carbohydrate-rich diets in individuals at MO and, to a lesser extent, at RC. The results support the emerging view that food production through plant cultivation was practised along the Atlantic Forest coast contemporaneously to the emergence of farming villages in the Andes [16] and Amazonia [8,25], and early plant cultivation in the La Plata Basin [1].

### 1.1. The *sambaquis* of southern Brazil

**Figure 1.**
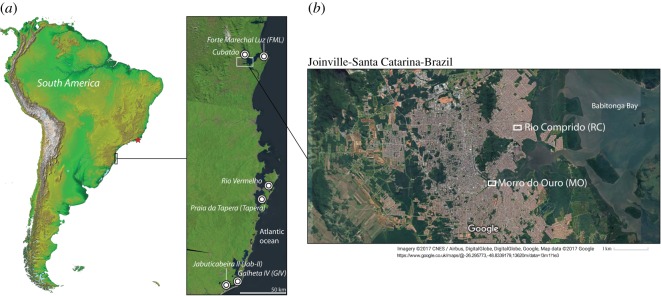
(*a*) Localities of Middle and Late Holocene coastal sites mentioned in the text. The red star indicates the geographical location of Middle Holocene coastal sites (Forte, Corondó, both in Saquarema region) with possible evidence of food production [26–28]. (*b*) Locations of Morro do Ouro (MO) and Rio Comprido (RC) in Joinville.

The northern coast of Santa Catarina state has the highest concentration of *sambaquis* along the Brazilian coast (figure 1*a*; electronic supplementary material, Methods) [29]. Hundreds of sites are distributed around Babitonga Bay, where recent population growth and urban development have been putting increasing pressure on this unique archaeological heritage. The archaeological information contained within some of these sites, such as MO and RC (figure 1*b*), has survived thanks to preventive archaeological investigations and private collections [30–32], along with previous scientific studies [21,23,33] and curation at the Museu Arqueológico de Sambaqui de Joinville.

Morro do Ouro (MO) has been a key site in discussion of population density, health and disease, and cultural and dietary variability in the Atlantic Forest coast during the Middle Holocene [23]. Preventive archaeological excavations from the second half of the twentieth century report great amounts of terrestrial and marine faunal remains, artefacts, domestic structures and human burials [30–32]. Faunal remains include molluscs (e.g. *Anomalocardia flexuosa*, Ostreidae, Mytilidae), fish (e.g. *Mugil* sp., *Micropogonias furnieri*, Centropomidae, Tetraodontidae, Sciaenidae, Ariidae), and terrestrial mammals (e.g. *Cuniculus paca*, *Tayassu pecari* [31]; J. Ferreira 2018, personal communication), but detailed taxonomic and quantitative information is lacking. Polished stone tools have been found, and charred plant remains (palm fruit) have also been reported in some archaeological deposits [32]. A total of 116 human burials were recovered at MO [30,31,33] and new radiocarbon measurements on human individuals (electronic supplementary material, table S1) reveal that the site was occupied between *ca* 4824–4527 and *ca* 4510–4101 cal BP. Analyses of micro-remains from dental calculus identified starch grains compatible with sweet potato (*Ipomoea batatas*), yam (*Dioscorea* sp.) and Araceae among others as yet unidentified [21]. However, the contribution of plant resources to individual diets was unclear until now.

Rio Comprido (RC), located *ca* 4 km from MO, was first excavated in 1969. A variety of lithic artefacts (choppers, flaked and semi-polished axes, stone sculptures), charcoal and faunal remains were extracted from the deposit [34] but, as for MO, there is a general lack of taxonomic and quantitative information on food remains. A total of 67 human burials were excavated [33]. Based on field reports, the burials were distributed in at least two funerary packages representing two distinct occupational phases: an earlier phase, RCI (*ca* 5642–5438 to *ca* 4800–4374 cal BP) and a later phase, RCII (*ca* 4051–3712 to *ca* 3608–3380 cal BP) [33,34], as further demonstrated by direct ^14^C measurements on human individuals (electronic supplementary material, table S1).

## 2. Material and methods

Detailed materials, sample preparation methods and results are reported in electronic supplementary material (Methods).

### 2.1. Sex, age and oral health markers

In this study, a total of 70 individuals were analysed. We applied morphological analyses, including sex and age determinations, as well as oral pathology analyses on 28 individuals from RC (divided in two chronological phases: RCI, *n* = 16; RCII, *n* = 12) and 42 from MO. We used 11 markers grouped into three categories (caries, periodontal disease and dental wear).

### 2.2. Stable isotope analysis and Bayesian mixing model

Stable isotope analysis was performed on 36 individuals, 16 from RC (RCI, *n* = 9; RCII, *n* = 7) and 20 from MO. Individual ribs, cranial fragments and bulk dentin were sampled for stable isotope analysis of carbon (δ^13^C_col_) and nitrogen (δ^15^N_col_) of bulk collagen. Teeth were also selected for apatite stable carbon (δ^13^C_ap_) isotope analysis from 12 and 8 individuals from MO and RC, respectively. The proportional contribution of different food sources to human diet at MO and RC was estimated using Bayesian mixing models in FRUITS 2.1.1 [35] to account for multiple dietary sources, macronutrient fractions and routing, and uncertainties in dietary inferences. While for bone collagen the only dietary proxies used in model estimations were δ^13^C_col_ and δ^15^N_col_ values, for teeth we explored collagen data alone (δ^13^C_col_ and δ^15^N_col_) and collagen data combined with apatite (δ^13^C_col_, δ^15^N_col_ and δ^13^C_ap_). Uncertainty of dietary proxies was set at 0.5‰. Three food sources with their respective macronutrient compositions were considered: terrestrial mammals (protein, lipids), fish (protein, lipids) and plants (protein, carbohydrates).

### 2.3. Radiocarbon age

To refine the chronology of Morro do Ouro and Rio Comprido, new ^14^C dates were obtained on selected individuals from each site, from different depths in the sedimentary record. Samples were analysed at Beta Analytic and at the University of Arizona AMS Facility using accelerator mass spectrometry. The ^14^C dates were calibrated (BP) using SHCal13 in OxCal v. 4.3 [36]. The Bayesian mixing model estimated the average relative contribution of marine carbon (%) to collagen carbon, which was then used to correct the radiocarbon dates for the marine reservoir effect for each individual. We adopted an average marine radiocarbon reservoir correction value (Δ*R*) of 23 ± 52 for the study area according to the data obtained on the southern Brazilian coast [37] and generated by http://calib.org/marine/.

## 3. Results

### 3.1. Radiocarbon determination

The consumption of marine resources had a measurable impact on the absolute ^14^C dates (electronic supplementary material, table S1). The Bayesian model (see below; electronic supplementary material, tables S6 and S7) estimated that carbon from marine organisms (e.g. fish) contributed from approximately 73.2 ± 3.6% (MO13) to approximately 3.7 ± 2.7% (MO59) to the carbon collagen of dated individuals, resulting in calibrated dates (2*σ*) older by up to approximately 377 years (MO13). The vertical distribution of ^14^C dates from RC confirms the sedimentary and archaeological evidence for at least two main phases of occupation, RCI (5642–5438 to 4800–4374 cal BP) and RCII (4051–3712 to 3608–3380 cal BP). These phases are separated by a stratigraphic deposit with no human burials. A possible much later phase of occupation may have occurred, as indicated by the date provided by burial RC4A which was recovered at a depth of 0–1 m but dated to 925–699 cal BP. However, this needs to be clarified with further studies.

Contrary to RC, the ^14^C dates from Morro do Ouro (4824–4527 to 4510–4101 cal BP) show no relation to their depth in the deposit. This could be related to several, not mutually exclusive factors such as spatial variability of site formation processes [38], post-depositional deformation [39] and secondary burials [14]. Nevertheless, considering that most of the dates overlap each other, it is possible that the funerary deposit at MO was formed relatively quickly. Therefore, we consider the individuals exhumed from MO as a single group.

### 3.2. Oral health

Using 11 oral health markers, a total of 1826 alveoli and 1345 teeth were examined from 70 individuals (see electronic supplementary material, Methods). Age and sex determinations were possible for approximately 75% of all analysed individuals (electronic supplementary material, table S2), with the majority of these determined to be male. As the sample size did not allow for statistical comparison by age and sex within sites, only between-site comparisons were made. Similar age distributions were seen at MO and RCII, represented primarily by middle adults (MA; 30–49 years) and young adults (YA; 20–29 years). A higher relative frequency of YA was found at RCI, followed by MA; however, these differences were not statistically significant (see electronic supplementary material, Methods for an expanded explanation). As such, the differences in oral pathology seen across the sites most likely reflect variability in diet and nutrition, rather than between-population differences in age or sex distribution [40].

The frequency of caries (electronic supplementary material, table S3) ranged from 7.6% (RCI) to 13.2% (MO), with statistically significant differences seen only between MO and RCI (*p* = 0.0052; differences between RCI and RC2, and between RC2 and MO were not significant). The frequencies of caries were higher than expected for most hunter-gatherers or fishermen [41]. By contrast, ante-mortem tooth loss (AMTL) reaches 6.2% (RCI), 2.4% (MO) and 1.1% (RCII), with statistically significant differences seen between RCI and RCII (*p =* 0.001) and RCI and MO (*p =* 0.0004).

Some differences were observed in caries depth between sites, but these were all statistically indistinguishable. Enamel caries ranged from 55% (RCII) to 80% (RCI), while the frequency of dentin caries, a ‘robust’ marker of cariogenicity [42], was higher in MO (23.1%) and RCII (20%), compared to RCI (15%). Pulp caries, which correspond to lesions reaching the pulp chamber producing necrosis, ranged from 5% (RCI) to 13.2% (MO) and 25% (RCII).

**Figure 2.**
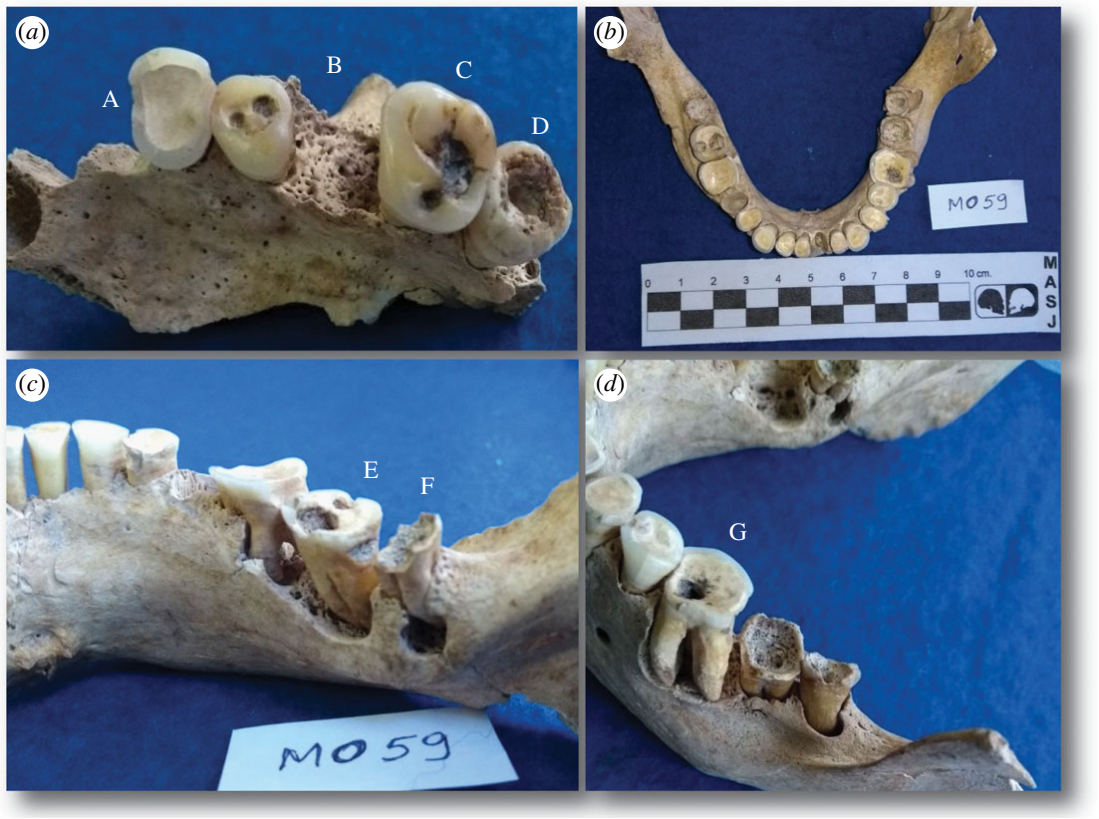
Oral pathology of individual MO59 (female, YA). (*a*) Left maxilla: A, dental wear; B, ante-mortem tooth loss; C, occlusal–dentin caries and pit caries; D, occlusal–pulp caries. (*b*) Mandible, occlusal view. (*c*) Mandible, lingual view on right side: E, M2: occlusal caries and related abscess and cervical caries; F, M3: gross–gross caries and related abscess. (*d*) Mandible, buccal view on left side: G, M1: occlusal pulp caries, abscess and alveolar resorption.

Similarly, caries type varied considerably between sites, but statistical differences were only observed for cervical (extra-occlusal) lesions (electronic supplementary material, table S3). The frequency of occlusal caries was generally high in all groups, ranging from 53.7% (MO) to 70% (RCI). The higher frequency in RCI, which has mainly enamel caries, confirms the chronicity of carious lesions. By contrast, MO had the highest frequency of extra-occlusal caries (including approximal, smooth surface and cervical lesions), and this was statistically significant when compared with RCII (*p* = 0.0123). Carious lesions have been associated with diets rich in fermentable carbohydrates [43–45] and free sugar including honey and syrups [46], with the frequency of deep caries increasing in retentive and non-retentive surfaces with exposure to cariogenic foods [47]. Diets higher in cariogenic foods typically have an increased frequency of extra-occlusal caries [42] and cavities on smooth or non-retentive tooth surfaces [48,49]. Therefore, it is likely that people at MO had a more cariogenic and refined (by mechanical fractionation and/or cooking by gelatinization) diet compared to those at RCII [40,50], as further corroborated by a significantly higher frequency of cervical lesions at MO (*p* = 0.0230) (figure 2*a–d*). Finally, caries linked to dental wear (occlusal wear, pulp exposure and dentin caries [40]) occurred more frequently in individuals from RCII (25%) compared to both MO (7.4%) and RCI (5%). However, there were no significant differences between sites.

Dental calculus, alveolar resorption [42] and dental wear indexes [51] were statistically indistinguishable across the three populations (*p* = 0.367, *p* = 0.437, *p* = 0.164; electronic supplementary material, table S4). Regarding prevalence (electronic supplementary material, table S5), MO showed the highest prevalence of carious lesions and AMTL, but differences were statistically significant only for periapical lesions, which were also more frequent in MO (41%) when compared to RCI (*p* = 0.0425).

### 3.3. Stable isotopes and Bayesian mixing models

Dietary estimations based on stable isotopes from teeth and bone were in good agreement with inferences from oral pathology. Bayesian mixing models quantified the relative caloric contribution (%) of three main food sources: plants, marine–estuarine fish and terrestrial mammals. The models also provided the relative caloric contribution of food macronutrients (e.g. protein), and the main source of dietary proteins based on the caloric contribution of food sources to nitrogen isotope values [35] (electronic supplementary material, tables S6 and S7). More accurate dietary reconstructions would have been achieved if isotopic baselines were available for each site, and their macronutrients analysed for stable carbon and nitrogen isotope composition. Moreover in this study, isotope signatures from teeth represent average individual diets of the first 2 to 20 years. Model estimations were based on teeth collagen and apatite, and on teeth collagen only. However, the generated estimates from these two models generally deviated less than 10% for all food sources, indicating that the model outputs were robust under distinct parameters. Some estimates deviated more than 10%, but these were limited to three individuals from MO (MO22, MO44, MO60) where the relative contributions of terrestrial mammals and plants to dietary calories could not be statistically resolved.

**Figure 3.**
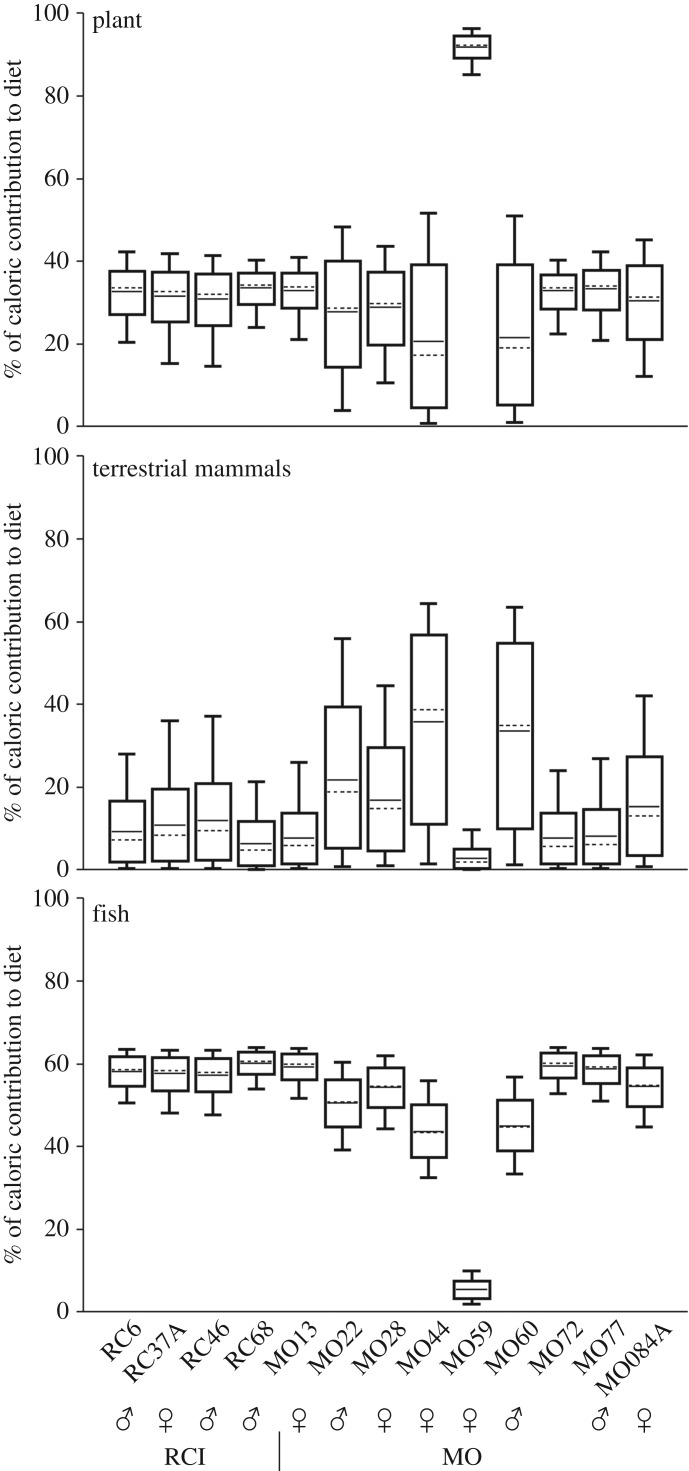
Individual model estimates of caloric intake for MO and RC (RCI) based on teeth enamel (δ^13^C_ap_) and dentin collagen (δ^13^C_col_ and δ^15^N_col_) isotope data. The box plot represents 68% (box) and 95% (whiskers) of credible intervals, with average (horizontal line) and median (dotted line).

Although the differences between teeth model estimates were relatively small, the model integrating collagen and apatite provided the most accurate outputs, with uncertainties associated with individual estimates generally less than 10%. Under this model, fish contributed to the majority of caloric intake at MO (approx. 5–60%; average 48 ± 16%), followed by plants (approx. 21–92%; average 36 ± 20%) and terrestrial mammals (approx. 3–36%; average 17 ± 11%). The wide credible intervals and standard deviations around the averages indicate that individual diets were remarkably variable at MO (figure 3). An example is offered by individual MO59, a young woman who obtained approximately 92% of her dietary calories from plants, and was affected by the highest number of carious lesions in the whole assemblage (figure 2*a*–*d*). Isotope data from teeth at RCI also suggest that fish was the main source of dietary calories (approx. 57–60%; average 58 ± 1%), followed by plants (approx. 31–34%; average 32 ± 1%) and terrestrial mammals (approx. 6–12%; average 10 ± 2%), but the much narrower credible intervals reflect more accurate estimations and less diversified diets (figure 3; electronic supplementary material, table S6).

For both sites, the main source of protein was fish, ranging from approximately 33 to 89% at MO and from approximately 87 to 90% at RCI. The large variability at MO was again largely due to MO59, which obtained approximately 59% of dietary proteins from plants, and the remaining approximately 33% and approximately 8% from fish and terrestrial mammals, respectively. In general, protein intake was high at RCI (approx. 22%) and MO (approx. 20–22%), with the exception of MO59 (approx. 5.4%), and compatible with values reported for other hunter-gatherers (19–35%) [52].

**Figure 4.**
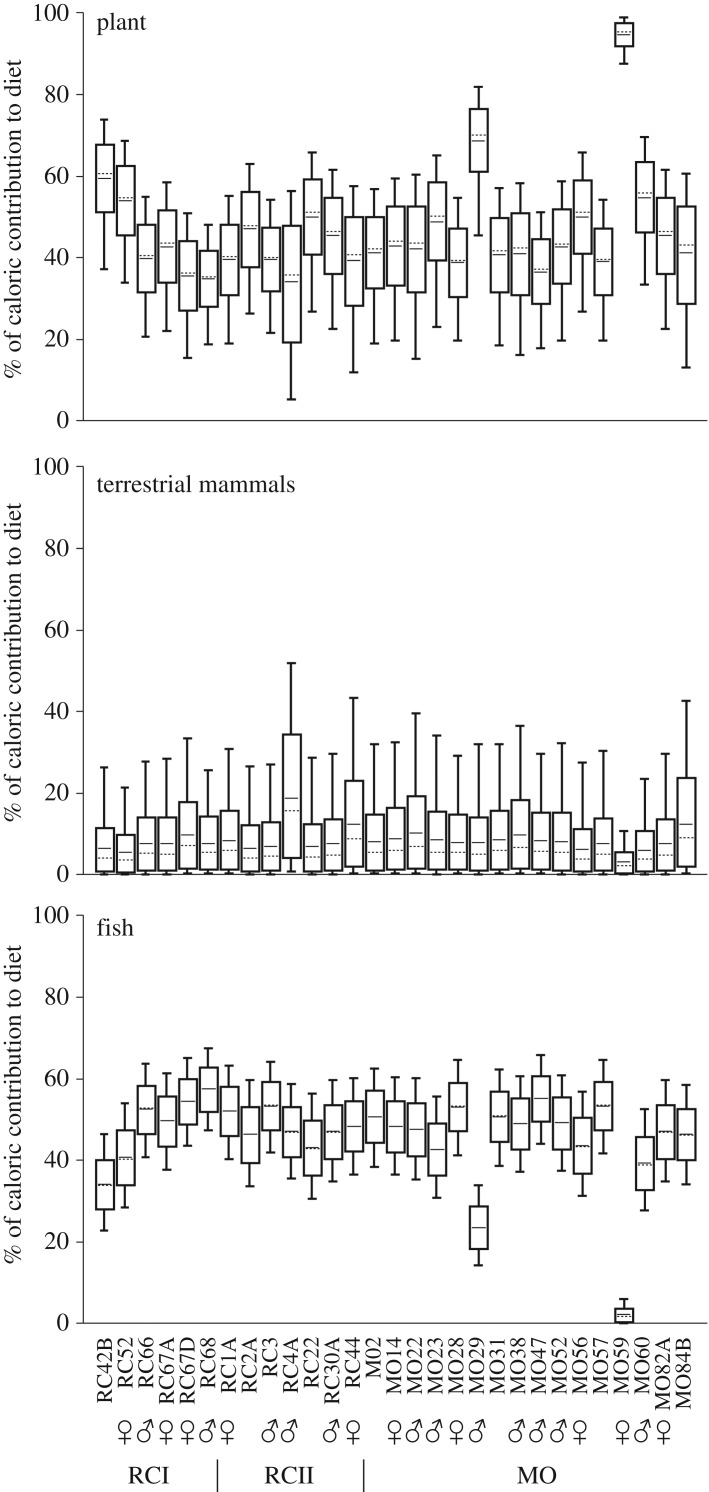
Individual model estimates of caloric intake for MO and RC (RCI and RCII) based on bone collagen isotope data (δ^13^C_col_ and δ^15^N_col_). The box plot represents 68% (box) and 95% (whiskers) of credible intervals, with average (horizontal line) and median (dotted line).

Dietary estimations based on bone collagen (figure 4; electronic supplementary material, table S7) suggest that plants provided the majority of dietary calories at MO (approx. 37–97%; average 48 ± 14%), along with fish (approx. 2–55%; average 44 ± 13%), and to a lesser extent terrestrial mammals (approx. 3–12%; average 8 ± 2%). Worth noting are two outliers, MO59 (female) and MO29 (male), with approximately 92% and approximately 70%, respectively, of their dietary calories coming from plants. As observed from teeth outputs, the wide range of estimated food sources highlights the diversified nature of individual diets at MO. Conversely, the model points to fish as the main source of dietary calories at RCI (approx. 34–57%; average 48 ± 8%) along with plants (approx. 35–59%; average 44 ± 9%), and to a lesser extent terrestrial mammals (approx. 5–10%; average 7 ± 1%). Similar estimates were obtained for RCII, where diet was dominated by fish (approx. 43–53%; average 48 ± 3%), followed by plants (approx. 34–50%; average 42 ± 5%) and terrestrial mammals (approx. 7–19%; average 10 ± 4%). In general, the food sources were statistically distinguishable within the 68% confidence interval for all the sites. Comparisons between bone and teeth estimates for the same individuals showed very little differences under the same model parameters (collagen only).

The main source of dietary proteins at MO was fish (approx. 58–84%), except for MO59, who obtained most of her dietary proteins from plants (approx. 82%). Fish was also the dominant source of proteins at RCI (approx. 66–85%) and RCII (approx. 74–83%). Protein intake estimated from bone collagen was higher compared to teeth, ranging from approximately 12 to 41% in MO, approximately 31 to 42% in RCI, and approximately 37 to 40% in RCII. These estimates are consistent and slightly higher than values observed for some hunter-gatherers [52].

**Figure 5.**
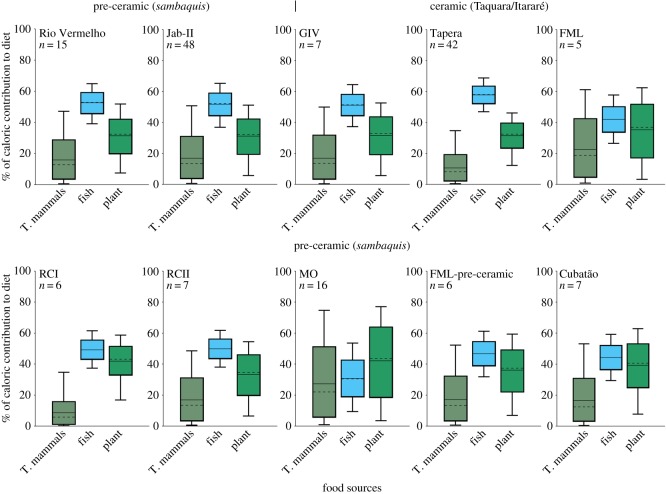
Model estimates of average (and standard deviation) caloric intake for MO and RC (RCI and RCII), and other pre-ceramic and ceramic sites discussed in this paper based on average and standard deviation of bone collagen isotope data (δ^13^C_col_ and δ^15^N_col_). The box plot represents 68% (box) and 95% (whiskers) of credible intervals, with average (horizontal line) and median (dotted line).

Model estimations based on the average δ^13^C_col_ and δ^15^N_col_ values of pre-ceramic and ceramic populations from the southern Atlantic Forest coast of Brazil indicate that fish was the main source of dietary calories, variably followed by plants and terrestrial mammals (figure 5), in agreement with previous ethnographic studies showing that animal proteins and lipids provide the dominant source of energy to hunter-gatherers [53,54]. Within this regional context, MO stands out in regard to its high dietary dependence on plants, followed by other groups (RCI, Forte Marechal Luz (FML), Cubatão), all in Babitonga Bay.

## 4. Discussion

The relative frequency and patterns of caries reveal distinct dietary behaviours and potentially also food preparation techniques between groups from RC and MO. The high proportion of chronic or static caries at RCI suggests a less cariogenic diet compared to RCII and MO, and it is possibly associated with a more alkaline salivary pH, and/or phosphate and calcium content in marine-based diets [55,56]. By contrast, the higher frequency of deep and extra-occlusal caries at RCII, and notably at MO, points toward the pervasive and persistent consumption of cariogenic and processed carbohydrates [45,57].

Cervical caries are the most common type of extra-occlusal caries in MO (29%) and have been associated with frequent consumption of sucrose and solid fermentable starches [45,57], high concentrations of salivary lactobacilli [49,58], age and deposition of cervical calculus with gingival recession [57,59]. Frequencies of cervical caries around 16.0% were reported in Pleistocene hunter-gatherers from North Africa, and interpreted as the first signs of systematic harvesting and storage of wild plant foods rich in carbohydrates [60]. In Andean agriculturalists, cervical caries (up to 30%) were attributed to the consumption of fermentable beverages prepared with manioc, maize and other starch-rich foods [41]. Previous studies have shown that sucrose, starch with sucrose, fructose and dextrose, in decreasing order, stimulate the production of smooth surface caries and cervical caries, while high amounts of maltose and starches led preferentially to cervical caries [43,57]. Therefore, diets at MO were probably richer in fermentable carbohydrates than those at RC, as well as in relation to diets of ancient occupants of other *sambaquis* [41,61], and comparable with diets of some agriculturalists [41].

Dental wear indexes in RC and MO were, by contrast, the lowest among several *sambaqui* groups studied elsewhere [62]. Nevertheless, diet in RCII seems to have been more abrasive than that at MO, which was the site with the lowest sand/grit content in dental calculus among four pre-ceramic and ceramic sites [21]. Regardless of the quantity of sand/grit in dental calculus, its presence suggests direct roasting of processed food over charcoal or cooking in earth ovens [33], and this seems to be confirmed by the presence of stone vessels and grindstones at MO, possibly used in flour making [21,31,32], although we cannot rule out the effect of taphonomic agents [21]. The oral pathologies observed in MO thus far suggest a highly cariogenic and more processed diet, compared to RCI and RCII.

Dietary inferences from oral pathologies are broadly supported by stable isotope data. Bayesian estimations indicate that plants, marine–estuarine fish and terrestrial mammals contributed to different extents to individual diets, but a higher dependence on plant resources took place at MO between *ca* 4824–4527 and 4510–4101 cal BP. Although more accurate dietary reconstructions would benefit from the development of isotopic baselines for each site, our comparative analysis across distinct coastal populations shows that plant consumption at MO was substantially higher, and above the values expected for hunter-gatherers [53]. Some individuals from MO (e.g. MO59) who relied fundamentally on plant resources since childhood could be interpreted as non-locals [63,64], perhaps absorbed in the context of post-marital residence practices [65]. Population exchange between coastal fishing people and groups engaged in plant cultivation may have facilitated the spread of ideas, transferred ecological knowledge, biological materials and economic strategies. As such, differential interpopulation interactions could explain the distinct degree of plant dependence among *sambaqui* groups in this region (figure 5).

Results from MO are directly supported by the presence of starches and phytoliths consistent with tuber crops, such as yams (*Dioscorea* sp*.*), sweet potato (*Ipomoea batatas*) and Araceae, in dental calculus of the same individuals [21]. Artefactual evidence such as stone tools for plant processing also indirectly confirms the investment in plant exploitation and preparation. Such a level of plant dependence would probably require some kind of plant management, possibly through cultivation, to guarantee long-term and predictable returns. Conceivably, other root crops such as manioc (*Manihot* sp.), which has been associated with slash-and-burn cultivation [13], and fruits reported in South American tropical forests from the Early Holocene could have featured on the menu of this group [3,66,67]. Interestingly, abrupt increases in carbonized particles associated with extensive palaeofires have been detected in sediments from the Atlantic Forest coast and the southern Brazilian highlands dated from 10 400 until 3600 cal. BP [68,69]. These fires took place in the context of a wetter climate and some are believed to be anthropogenic in origin, potentially signalling the onset of slash-and-burn cultivation in the region from the Middle Holocene [70].

South America is a polycentric cradle of early food production. However, the nature, time and space of early plant cultivation are still being mapped. The dietary evidence discussed in this paper suggest that food production may have taken place in this region during the Middle Holocene, contemporaneous with the shift to horticulture in Amazonia [8,13,25] and the earliest evidence for plant cultivation in La Plata Basin [1,71,72]. Plant cultivation has been postulated for other groups along the Atlantic Forest coast during the Middle Holocene [10,26–28], reinforcing the contribution of *sambaqui* archaeological evidence for documenting early food production in the Americas.

## 5. Conclusion

The Neotropics is one of the world's centres of early food production, but the nature, time and place of early plant cultivation are still matters of debate. The Atlantic Forest coast has been largely peripheral in this narrative despite its unique biodiversity and archaeological records of dense human occupation since the Middle Holocene. Our study challenges this traditional view. We report on oral pathology and stable isotope evidence for carbohydrate-rich diets among hunter-fisher-gatherers in southern Brazil at *ca* 4500 cal BP. The high consumption of cariogenic and carbohydrate-rich food suggests that permanent populations subsisting on a mixed economy possibly cultivated plants along this narrow coastal strip.

## Supplementary Material

Supplementary Material_Methods

## Supplementary Material

Supplementary Material_Tables

## Supplementary Material

Supplementary Material_Database Oral Health Markers

## Data accessibility

Data supporting this paper can be found in the electronic supplementary material. These include the stable isotope and radiocarbon data, the results of oral health studies and results of the Bayesian mixing model.
