# Supplementary material for: Middle Holocene plant cultivation on the Atlantic Forest coast of Brazil?
Source: R Soc Open Sci. 2018 Sep 5;5(9):180432. doi: 10.1098/rsos.180432 (PMC6170589; doi:10.1098/rsos.180432)
Supplement: Supplementary Material_Methods [file rsos180432supp1.docx]

**Middle Holocene plant cultivation on the Atlantic Forest coast of Brazil?**

Luis Pezo-Lanfranco^1^, Sabine Eggers^1,2^, Cecilia Petronilho^1^, Alice Toso^3^, Dione Da Rocha^4^, Matthew Von Tersch^3^, Adriana M.P. dos Santos^5^, Beatriz Ramos da Costa^5^, Roberta Meyer^5^, André Carlo Colonese^3^

^1^ Laboratório de Antropologia Biológica, Departamento de Genética e Biologia Evolutiva. Instituto de Biociências – Universidade de São Paulo. Rua do Matão 277, 05508-900, Cidade Universitária USP, São Paulo, Brazil

^2^ Naturhistorisches Museum Wien, Anthropologische Abteilung, Burgring 7, 1010 Vienna, Austria

^3^ BioArCh, Department of Archaeology, University of York, York, YO10 5DD, United Kingdom.

^4^ Universidade da Região de Joinville, Mestrado em Patrimônio Cultural e Sociedade, Rua Paulo Malschitzki 10, Zona Industrial Norte, 89219-710, Joinville, Santa Catarina, Brazil

^5^ Museu Arqueológico de Sambaqui de Joinville, Rua Dona Francisca 600, Centro, 89201-250, Joinville, Santa Catarina, Brazil

**Supporting Information**

**SI Methods**

**Morro do Ouro.** Morro do Ouro (MO) is located on the Cachoeira river that drains into Babitonga Bay. The original dimensions of the site are unknown. During the earliest excavations the site measured 140 x 70 x 28 m [[1]](https://paperpile.com/c/S9ddCY/OE7zw), but by the beginning of this century it had been extensively damaged, and today it measures 90 x 60 x 13 m [[2]](https://paperpile.com/c/S9ddCY/eOrVg). From the second half of the 20^th^ century, several preventive archaeological excavations recovered a great amount of artifacts, structures and human burials [[1,3,4]](https://paperpile.com/c/S9ddCY/jSpRo+CBl1s+OE7zw). Tiburtius [[1]](https://paperpile.com/c/S9ddCY/OE7zw) reported zooliths (stone zoomorphic sculptures typically associated with *sambaquis* [[5]](https://paperpile.com/c/S9ddCY/Ld4ym)), projectile points and other tools and artifacts (made from mammal bones and teeth, and mollusk shells), quartz crystals and ochre pigments as funerary offerings. Lithic artifacts included *quebra-coquinhos*, chopping tools and flaked stone axes. Beck [[3]](https://paperpile.com/c/S9ddCY/jSpRo), who only excavated 24 m^2^ of the north face of the mound, reported marine and terrestrial faunal remains, occupational surfaces, polished stone tools, shell artifacts and human burials. Goulart [[4]](https://paperpile.com/c/S9ddCY/CBl1s) excavated 1200 m^2^ and reported finding charcoal, polished stone axes, weights for fishnets, hearth remains and domestic areas. Faunal remains included mollusks (carib pointed venus, oysters, mussels), fish (e.g. mullet, snook, whitemouth croaker, puffer fish, drumfish, catfish), and terrestrial mammals (e.g. lowland paca, white-lipped peccary) [[1]](https://paperpile.com/c/S9ddCY/OE7zw); *Ferreira personal communication*), but detailed taxonomic and quantitative information is lacking. Plant remains were also recorded, including charred seeds of *jerivá* palm or palm fruits (*Syagrus romanzoffiana*) [[3,4]](https://paperpile.com/c/S9ddCY/jSpRo). Analyses of micro-remains from dental calculus detected starch grains compatible with sweet potato (*Ipomoea batatas*), yam (*Dioscorea* sp.) and Araceae, among other unidentified traces [[6]](https://paperpile.com/c/S9ddCY/QjBo2). A total of 116 human burials were recovered at MO [[1,3]](https://paperpile.com/c/S9ddCY/jSpRo+OE7zw). Some of them contained mammal vertebrae and teeth, lithic artifacts, ochre and quartz as funerary offerings [[1,4]](https://paperpile.com/c/S9ddCY/CBl1s+OE7zw). Radiocarbon dates are reported for a few individuals by Wesolowski [[6,7]](https://paperpile.com/c/S9ddCY/xfR1s+QjBo2), providing ages of 4,030 ± 40 BP (MO28), 3,870 ± 40 BP (MO80), 4,300 ± 50 BP (MO31).

**Rio Comprido.** Rio Comprido (RC) is located near Rio Comprido river, in the city of Joinville. The site, initially reported as a mound of 13 m in height and 60 m in diameter, was first excavated in 1969. The chronology of Rio Comprido is vaguely offered by Prous and Piazza [[8]](https://paperpile.com/c/S9ddCY/4rcbi) for deposits at different depths spanning a time interval between approximately 4,170 and 4,865 BP. Martin et al. [[9]](https://paperpile.com/c/S9ddCY/wPBPD) report a ^14^C date of 4,560 ± 110 BP that appears consistent with Prous and Piazza’s preliminary chronological interval. Lithic artifacts (choppers, flakes and semi-polished axes), charcoal, faunal remains, and pieces of one zoolith were extracted from the pluristratigraphic deposit [[8]](https://paperpile.com/c/S9ddCY/4rcbi), but no detailed taxonomic and/or quantitative information on food remains is available for Rio Comprido. A total of 67 human burials were excavated [[7,8]](https://paperpile.com/c/S9ddCY/4rcbi+xfR1s). Based on field reports, the burials were distributed in at least two funerary packages that possibly represent two distinct occupational phases: an earlier phase (RCI), located at a depth of 2.70 to 6.50 m, and a later burial phase (RCII) between 0.90 and 1.70 m deep [[7]](https://paperpile.com/c/S9ddCY/xfR1s).

**Sample selection**. Previous pathological and stable isotope studies have been performed on some of the individuals analysed in this work [[6,10,11]](https://paperpile.com/c/S9ddCY/QjBo2+4mkIk+N3zq7), but here we expanded the osteological record to integrate a more detailed oral pathology approach on recently dated individuals coupled with new stable isotope analysis of bone and dentin collagen (δ^15^N and δ^13^C_col_), and tooth enamel carbonate (δ^13^C_ap_). The morphological analyses included sex and age determinations as well as oral pathology analyses of 70 individuals: 28 from RC (divided in two chronological phases: RCI, n = 16; RCII, n = 12) and 42 from MO. Stable isotope analysis was performed for 36 individuals: 16 from RC (RCI, n=9; RCII, n=7) and 20 from MO.

**Sex and age determination.** Sex determinations were made for 60 adult individuals based on pelvic and cranial morphology [[12]](https://paperpile.com/c/S9ddCY/LLwAJ) (table S2): 28 from Rio Comprido (divided in two chronological phases: RCI, n = 16; RCII, n = 12) and 42 from Morro do Ouro (MO). Because morphological sex estimation of juveniles is problematic, they were all classified as sex-undetermined. Age at death in adults was estimated by pubic symphysis morphology, 4^th^ rib surface, auricular surface, and cranial suture closure. In subadults, age at death was estimated based on tooth formation and eruption charts, vertebral development and epiphyseal closure (all methods in [[12]](https://paperpile.com/c/S9ddCY/LLwAJ)). Given the uncertainties of exact age determination for fragmented skeletons, such individuals were classified in broad age-categories [[12]](https://paperpile.com/c/S9ddCY/LLwAJ). As our sampling focused on the best-preserved individuals to guarantee the best possible record of oral pathology and isotope values, it is possible that our sample is not demographically representative [[13]](https://paperpile.com/c/S9ddCY/bbfbK). However, the distribution of the analyzed groups by age classes (*Tab. S2*) does not show significant differences (Pearson Chi Square: *X*^2^ = 9.494; df = 8; *p =* 0.302; Jonckheere-Terpstra Test: Observed J-T Statistic = 829.000; *p =* 0.068).

**Oral health markers.** Oral pathology markers are informative about the various dimensions of dietary behaviors and provide valuable data about the cariogenicity of diet, quality and quantity of carbohydrates in the diet and physical features of food (related with preparation and/or conservation techniques [[14,15]](https://paperpile.com/c/S9ddCY/qvgW8+4nAh7). We used 11 oral health markers grouped into caries, periodontal disease and dental wear categories [[16,17]](https://paperpile.com/c/S9ddCY/9uFad+9T3MP) (tables S3, S4 and S5).

**1-** Caries frequency (number of carious teeth by total number of preserved teeth); **2 –** Antemortem tooth loss (AMTL) frequency (number of AMTL, by total number of preserved alveoli); **3 -** Frequency of caries by depth (number of carious lesion by tissue affected per total number of carious teeth). Scores: a) enamel caries; b) dentin caries; c) pulp caries; d) gross- gross caries; **4 -** Frequency of caries type (number of a certain type of carious lesion per total number of carious teeth). Scores: a) **Occlusal caries** usually affect sulci and fossae of premolar and molar occlusal surfaces. b) **Pit caries** affect vestibular sulci of lower molars, and the cingulum on the lingual surface of upper incisors and canines. c) **Smooth surface caries** **(B/L)** penetrate the smooth buccal or lingual surfaces. d) **Approximal caries** affect the contact area between two teeth. e) **Smooth surface caries (M/D)** affect the approximal surface below the contact point between two teeth in the coronal cervical third, not affecting the cementum-enamel junction. f) **Root surface caries and/or CEJ cari**es affect uncovered roots or near to the gum rim and the cementum-enamel junction. g) **Occlusal attrition, enamel edge chipping and caries** refer to the lesions associated with marginal enamel fractures caused by intense dental wear. h) **Occlusal wear, dentine caries and pulp exposure** refer to the lesions that affect the pulp through dental wear. j) **Gross-gross caries** refer to wide destruction of tooth crown or root remains. When more than one carious lesion appears in a tooth, the more “cariogenic” score was recorded. For instance: a tooth with two lesions, occlusal and approximal, was classified as Score 4 (approximal caries); **5-7:** Prevalence (number of individuals affected by the condition in one population) of caries, AMTL, periapical lesions. **8 -** Prevalence of periodontal disease: Frequency of individuals with alveolar resorption score >1 were considered as affected with periodotitis (a clinical stage with inflammatory and alveolar bone loss, mobility and periodontal bags); **9 -** Dental calculus index: calculus accumulation in a crown or root of a tooth. Scores: 1) scarce supra-gingival calculus (stain or calcified lines <1 mm of thickness); 2) abundant supra-gingival calculus (>1 mm of thickness); 3) Infra-gingival calculus (below the CEJ) of any thickness. Lingual and buccal surfaces were recorded and a tooth average was computed. The individual index is the average of all teeth recorded. The group index is the interindividual average; **10 -** Alveolar resorption index: bone resorption by alveolus Scores: 1) between 2 mm and less than ¼ of root length; 2) between ¼ and ½ of the root length; 3) more than ½ of the root length. Lingual and buccal surfaces were recorded and a tooth average was computed. The individual index is the average of all alveoli recorded. The group index is the interindividual average; **11 -** Dental wear index: Use an ordinal scale (scores 1 to 8) to quantify occlusal wear by incisors, premolars and molars. The individual index is the average of all teeth recorded. The group index is the interindividual average.

Although some oral health markers are age-related (periodontal disease, AMTL, dental wear), the progression of carious lesions (faster or slower) is more dependent on the relative virulence of the oral bacteria and the quantity and quality of carbohydrate consumed than age [[18]](https://paperpile.com/c/S9ddCY/fFGIj).

**Stable carbon and nitrogen isotope analysis of bone and dentin collagen.** Bone collagen samples for stable isotope analysis of carbon (δ^13^C_col_) and nitrogen (δ^15^N_col_) of bulk collagen (table S6) were extracted at the BioArCh facilities of the Department of Archaeology, University of York (UK). Bones were cleaned mechanically to remove the surface and the extraction followed a modified Longin method [[19]](https://paperpile.com/c/S9ddCY/Iavo2); details can be found in previous studies [[20]](https://paperpile.com/c/S9ddCY/H9QmK). In short, shards of bones (ca. 200 to 300 mg) were demineralized using 0.6 M HCl at 4 °C for several days, then rinsed with ultrapure H_2_O (milli-Q^®^) and gelatinized with 0.001 M HCl at 80 °C for 48 h. Samples were then ultrafiltered (30 kDa, Amicon^®^ Ultra-4 centrifugal filter units; Millipore, MA, USA), frozen and freeze dried. Collagen samples (1 mg) were combusted to obtain CO_2_ and N_2_ with a Sercon GSL system attached to a Sercon 20-22 mass spectrometer (Sercon, Crewe, UK). Standardization was carried out using IAEA 600 and IA-006 for δ^13^C, and IAEA-N-2 and IAEA-600 for δ^15^N. Precision was <0.2‰ (1σ) for both δ^13^C and δ^15^N values.

Collagen was also extracted from the dentin of 12 and 8 individuals from MO and RCI, respectively, for bulk stable carbon and nitrogen isotopes analysis at the Environmental Isotope Laboratory of the Department of Geosciences, University of Arizona (USA) (table S7). Each tooth was sectioned to separate enamel from dentin. Bulk dentin collagen was prepared using a method modified from [[21]](https://paperpile.com/c/S9ddCY/bxyqj). Samples were sonicated in deionized H_2_O, the outer layer was removed and then ground to < 1.0 mm particle size using a mortar and pestle. Approximately 100 - 500 mg dentin powder was treated with 0.5 M HCl for demineralization, followed by deionized water for neutralization and 0.1 M HCl for acidification. The resultant solids were gelatinized in 0.1 M HCl at 70 °C for 20 h. The final solution was filtered through a 0.45 μm glass microfiber filter, frozen and lyophilized. Collagen samples (1 mg) were combusted to obtain CO_2_ and N_2_ with a Costech EA system attached to a Finnigan Delta PlusXL mass spectrometer. Standardization was carried out using IAEA CH-7 and USGS-24 for δ^13^C, and IAEA-N-1 and IAEA-N-2 for δ^15^N. Precision was <0.2‰ (1σ) for both δ^13^C and δ^15^N values.

**Stable carbon isotope analysis of enamel carbonate.** Teeth were selected for apatite stable carbon (δ^13^C_ap_) isotope analysis from 12 and 8 individuals from MO and RC, respectively. Crown fragments containing only enamel were mechanically cleaned with scrapers, dental brushes, and nitric acid, and powdered with a carbide dental drill. Samples of approximately 20 mg were then immersed in 0.1 N C_2_H_4_O_2_ for 2 h and rinsed three times with distilled water. The enamel powder was allowed to react with H_3_PO_4_ and Ag at 70°C in a KIEL-III sample preparation system. The δ^13^C values of the resultant CO_2_ were measured using a Finnigan MAT-252 Isotope ratio mass spectrometer. Calibration of the isotope ratios were based on repeated measurements of NBS-19 and NBS-18 with a precision of ± 0.08 ‰ for δ^13^C (1σ). Cleaning and measurement procedures are based on Koch et al. [[22]](https://paperpile.com/c/S9ddCY/q2ci3).

All teeth sampled represent ages ranging from at least 2-11 years old (1^st^ permanent premolar crown develops between 2-6 years, whereas root develops between 6-11 years) to 10-20 years old (3^rd^ permanent molar crown develops between 9-12 years, whereas root develops between 12-20 years), from infantile to juvenile age-ranges [[12]](https://paperpile.com/c/S9ddCY/LLwAJ). Diet was estimated for teeth (δ^13^C_col_, δ^15^N_col_ and δ^13^C_ap_ values) and bone (δ^13^C_col_ and δ^15^N_col_ values) to explore differences in age, and because not all individuals were equally represented by these elements.

Results of stable isotope analyses are reported in δ-notation, where δ^13^C_col_, δ^13^C_ap_ and δ^15^N_col_ values are given as parts per mil (‰) deviations from the standards VPDB (Vienna Pee Dee Belemnite - PDB) for both δ^13^C_col_ and δ^13^C_ap_, and AIR (atmospheric N_2_) for δ^15^N_col_.

**Collagen extraction and stable isotope results**. Dentin collagen was successfully extracted from all individuals (n = 20), but only samples with the molecular characteristics of preserved collagen according to the literature [[23–26]](https://paperpile.com/c/S9ddCY/Q5j9K+5IBHb+2duKC+DDbk0) were included in this study (n = 13; 65 %). The δ^13^C_col_ and δ^15^N_col_ values of dentin ranged from -13.9 ‰ to -13.1 ‰ and from +14.5 ‰ to +15.7 ‰ in RC, whereas in MO the δ^13^C_col_ and δ^15^N_col_ values ranged from -12.9 ‰ to -21.3 ‰ and from +15.4 ‰ to +10.0 ‰, respectively. No significant differences were observed between the sites/series for both δ^13^C_col_ and δ^15^N_col_ values (*p* = 0.299 and 0.168, respectively). In general, no significant differences were observed between bulk dentin and bone δ^13^C_col_ and δ^15^N_col_ values for MO (n = 4; *p <* 0.05 for all tests). The small sample size from RC prevented testing differences between bone and dentin collagen for the same individuals. Finally, the δ^13^C_ap_ values ranged from -9.0 ‰ to -7.3 ‰ in RCI (n = 4) and from -12.3 ‰ to -7.4 ‰ in MO (n = 9), with no significant differences between the sites (*p* = 0.098).

Bone collagen was successfully extracted from 29 (93.5 %) out of 31 individuals. The wt % of C and N ranged from 16.2 % to 40.5 % and 5.5 % to 14.5 %, respectively, while the the C:N ratios ranged from 3.3 to 3.6. These values are in the expected range of preserved collagen [[23–26]](https://paperpile.com/c/S9ddCY/Q5j9K+5IBHb+2duKC+DDbk0). The δ^13^C_col_ and δ^15^N_col_ values of bone ranged from -11.4 ‰ to -15.2 ‰ and from +16.3 ‰ to +11.7 ‰ in RC, whereas in MO the δ^13^C_col_ and δ^15^N_col_ values ranged from -12.0 ‰ to -20.6 ‰ and from +14.5 ‰ to +7.0 ‰, respectively. No significant differences were observed between the sites for both δ^13^C_col_ and δ^15^N_col_ values (*p* = 0.261 and 0.326, respectively). Similarly, no significant differences were observed for δ^13^C_col_ and δ^15^N_col_ values between the earlier (RCI) and the later (RCII) phases of RC (*p* = 0.748 and 0.807, respectively). The δ^13^C_col_ and δ^15^N_col_ values did not differ significantly between sexes for either site (*p* < 0.05 for all tests).

**Statistical analysis.** Depending on variable type and distribution, descriptive and inferential statistical analyses of oral pathology markers were performed using U Mann-Whitney, Kruskal-Wallis, Chi-square, and Fisher exact tests with α = 0.05 using SPSS v. 18.0 package (IBM^®^). The δ^13^C_col_ and δ^15^N_col_ values (bone and dentin) and δ^13^C_ap_ values between sites, phases (RCI and RCII) and individuals (sex) were compared using one-way ANOVA (α = 0.05), after checking for normal distribution with the Shapiro-Wilk test for normality (α = 0.05). The δ^13^C_col_ and δ^15^N_col_ values from bone and dentin for the same individuals for MO were compared using a paired t-test (after checking for equality of variance with F test). Statistical tests were performed in PAST 3.x [[27]](https://paperpile.com/c/S9ddCY/HRXyL).

The proportional contribution of different food sources to human diet at MO and RC was estimated using a Bayesian mixing model in FRUITS 2.1.1 [[28]](https://paperpile.com/c/S9ddCY/9g9vV) based on dietary proxies (stable isotopes) of humans and food items. Unfortunately, the faunal remains recovered at MO and RC were not available for isotopic analysis. We therefore used the average δ^13^C_col_ and δ^15^N_col_ values of terrestrial mammals (-22.0 ± 1.2‰ and +8.4 ± 1.7‰ respectively, n = 14) published by Colonese et al. in 2014 (32). These consisted of Late Holocene herbivorous and omnivorous taxa from the Atlantic Forest coast of Brazil (e.g. peccary, lowland paca, brocket deer, howler monkey, six-banded armadillo). Their δ^13^C_col_ values indicate diets based on C_3_ plant ecosystems.

For fish we integrated two sets of samples to take into account fishing in marine and estuarine environments by groups at Babitonga Bay. The first set of samples includes the average δ^13^C_col_ and δ^15^N_col_ values (-11.4 ± 1.4‰ and +13.5 ± 2.0‰ respectively, n = 17) of modern (n = 10) and archaeological (n = 7) fish specimens published in Colonese et al. [[29]](https://paperpile.com/c/S9ddCY/F1nP4). These are specimens captured in the open sea and coastal bays of southern Brazil. The δ^13^C_col_ values of modern specimens were previously corrected for the Suess effect [[29]](https://paperpile.com/c/S9ddCY/F1nP4). A second set of samples corresponds to modern specimens (n = 5) recently captured in the estuary of Babitonga Bay [[30]](https://paperpile.com/c/S9ddCY/PBUhi). The δ^13^C and δ^15^N values of flesh were first corrected for the Suess effect (+ 2 ‰) using the δ^13^C value of the atmosphere in 2010 (-8.4 ‰) [[31]](https://paperpile.com/c/S9ddCY/BIzpD), and then converted to δ^13^C_col_ and δ^15^N_col_ values (-13.5 ± 1.5 ‰ and +11.1 ± 2.4 ‰ respectively) using offsets between flesh tissue and bone collagen of -2 ‰ and +1.5 ‰ respectively [[32,33]](https://paperpile.com/c/S9ddCY/W8VvG+o5zeQ). Babitonga Bay is currently an estuary under the influence of freshwater and seawater circulation, with stable carbon and nitrogen isotopic signatures which differ significantly [[34]](https://paperpile.com/c/S9ddCY/VfdO1). However, during the Middle-Late Holocene, the area was under a more direct input of oceanic circulation due to the high sea-level stand along the southern coast of Brazil [[35–37]](https://paperpile.com/c/S9ddCY/WRg45+xvJf3+FUI5L). Preliminary analyses of fish remains from MO and other *sambaquis* in Babitonga Bay reveal that fishing was predominantly practiced in marine and estuarine environments (*Fossile, Ferreira,* *personal communications*). This consequently legitimates the use of mixed marine and estuarine fish isotope values for dietary reconstructions at the inner sector of the bay. For plants we used the average δ^13^C and δ^15^N values of modern fruits (n = 30), roots (n = 5) and palm-heart (n = 13) (-29.2 ± 3.0 ‰ and +1.1 ± 2.0 ‰, respectively) collected in national parks in the southeastern Atlantic Forest between 2010 and 2012 [[38]](https://paperpile.com/c/S9ddCY/qqpJX). The δ^13^C values were corrected for the Suess effect (+ 2 ‰) using the δ^13^C value of the atmosphere in 2010 (-8.4 ‰) [[31]](https://paperpile.com/c/S9ddCY/BIzpD). Recent studies suggest that some later pre-ceramic coastal populations may have consumed maize [[39]](https://paperpile.com/c/S9ddCY/6c0XJ), a C_4_ plant. However the δ^13^C value single amino acids of these individuals indicate that maize or any other C_4_ plant, if consumed, had a negligible impact on human isotope composition [[29]](https://paperpile.com/c/S9ddCY/F1nP4). We have therefore excluded C_4_ plants from the model input.

For each food source the average δ^13^C and δ^15^N values of the nutrient fraction (protein, carbohydrate, lipids) was estimated using fractionations reported in [[40,41]](https://paperpile.com/c/S9ddCY/aIXX9+44KM8). For fish and terrestrial mammals the isotopic composition of their nutrient fraction was derived from their average δ^13^C_col_ and δ^15^N_col_ values using the following offsets: -2 ‰ (∆^13^C_protein-collagen_), -8‰ (∆^13^C_lipids-collagen_) and +2 ‰ (∆^15^N_protein-collagen_). For fish these are: -1 ‰ (∆^13^C_protein-collagen_), -7 ‰ (∆^13^C_lipids-collagen_) and +2 ‰ (∆^15^N_protein-collagen_). We used a conservative offset for lipid-collagen of -7 ‰, although previous studies suggest that for marine organisms the offset can be higher [[42]](https://paperpile.com/c/S9ddCY/ZESBI). For plants, the offsets are: -2 ‰ (∆^13^C_bulk-protein_) and +0.5 ‰ (∆^13^C_bulk-lipids_), and assuming that the δ^15^N value of plant protein was the same as the average bulk plant δ^15^N value. A conservative uncertainty of 1 ‰ was used for all offsets.

Since δ^13^C_ap_ values represent the total carbon in diet [[24]](https://paperpile.com/c/S9ddCY/5IBHb) we considered the weighted average of each food group (according to nutrient composition) as the source of the δ^13^C_ap_ values. For practicality, we considered nutrient concentrations (as dry weight) reported in [[40]](https://paperpile.com/c/S9ddCY/aIXX9). Instead, for collagen the nitrogen is assumed to derive exclusively from proteins (100%), but carbon isotopes can be variably sourced from carbohydrates and lipids during *de novo* synthesis of non-essential amino acids [[24,43–45]](https://paperpile.com/c/S9ddCY/SxgDb+qxYyT+8Sie7+5IBHb). We have therefore used estimations of carbon isotopes from protein and energy to bulk collagen of 74 ± 4 % and 26 %, respectively [[43]](https://paperpile.com/c/S9ddCY/SxgDb).

In order to integrate this “scramble” effect, a concentration-dependent and routed model was selected for the Bayesian estimations for both teeth and bone isotope data [[41]](https://paperpile.com/c/S9ddCY/44KM8). Lipids and carbohydrates are aggregated as energy in the model input. Diet-to-collagen and diet-to-apatite δ^13^C offsets (+5 ± 0.5 ‰ and +10.1 ± 0.5 ‰, respectively), and diet-to-collagen δ^15^N offset (+5.5 ± 0.5 ‰) were taken from [[43]](https://paperpile.com/c/S9ddCY/SxgDb), and these represent consensus values from distinct experimental studies. Finally, dietary estimations were constrained for a conservative acceptable range of protein intake of >5% and <45% of total calories according to physiological studies [[28]](https://paperpile.com/c/S9ddCY/9g9vV) (S8 Table).

Dietary estimations from MO and RC were compared with other sites from this region. The Bayesian mixing model was also applied to the average (and standard deviation) δ^13^C_col_ and δ^15^N_col_ values of other pre-ceramic coastal shellmound builders from Babitonga Bay (Cubatão) and its outermost part on the island of São Francisco (Forte Marechal Luz), from the central (Rio Vermelho) and southern (Jabuticabeira II) coast of Santa Catarina state dated between ~4,800 to 1,500 cal BP. We also included individuals who used ceramic vessels associated with the Taquara/Itararé tradition from the island of São Francisco do Sul (Forte Marechal Luz), and from the central (Tapera) and southern (Galheta IV) coasts of Santa Catarina state at ~1,000 cal BP [[29,46–49]](https://paperpile.com/c/S9ddCY/F1nP4+V5BKz+7lEQg+CIzPE+zgFJd). For sites outside Babitonga Bay (Forte Marechal Luz, Rio Vermelho, Jabuticabeira II, Tapera and Galheta IV), only fish published in Colonese et al. [[29]](https://paperpile.com/c/S9ddCY/F1nP4) were used.

**References**

1. [Tiburtius G. 1996 *Arquivos de Guilherme Tiburtius I*. Fundação Cultural de Joinville. Joinville: Museu Arqueológico de Sambaqui de Joinville.](http://paperpile.com/b/S9ddCY/OE7zw)

2. [Oliveira M. 2000, unpublished master dissertation Os Sambaquis da Planície costeira de Joinville, litoral norte de Santa Catarina: Geologia, paleogeografia e conservação in situ.](http://paperpile.com/b/S9ddCY/eOrVg)

3. [Beck A. 2007 *A variação do conteúdo cultural dos sambaquis do litoral de Santa Catarina*. Erechim-RS: Habilis.](http://paperpile.com/b/S9ddCY/jSpRo)

4. [Goulart M. 1980 Tecnologia e padrões de subsistência de grupos pescadores-coletores pré-históricos. Habitantes do Sambaqui Morro do Ouro – Joinville – Santa Catarina.](http://paperpile.com/b/S9ddCY/CBl1s)

5. [Prous A. 1974 Les sculptures préhistoriques du Sud-Brésilien. *bspf* **71**, 210–217.](http://paperpile.com/b/S9ddCY/Ld4ym)

6. [Wesolowski V, Ferraz Mendonça de Souza SM, Reinhard KJ, Ceccantini G. 2010 Evaluating microfossil content of dental calculus from Brazilian sambaquis. *J. Archaeol. Sci.* **37**, 1326–1338.](http://paperpile.com/b/S9ddCY/QjBo2)

7. [Wesolowski V. 2000 A prática da horticultura entre os construtores de sambaquis e acampamentos litorâneos da região da Baia de São Francisco, Santa Catarina: uma abordagem bioantropológica. unpublished master dissertation.](http://paperpile.com/b/S9ddCY/xfR1s)

8. [Prous A, Piazza W, editors. 1977 *Documents pour la préhistoire du Brésil Méridional 2: l’etat de Santa Catarina*. Cahiers d’Archéologie d’Amérique du Sud.](http://paperpile.com/b/S9ddCY/4rcbi)

9. [Martin L, Suguio K, Flexor JM. 1984 Informações adicionais fornecidas pelos sambaquis na reconstrução de paleolinhas de praia quaternária: exemplos da costa do Brasil. *Revista de Pré-História* **6**, 128–147.](http://paperpile.com/b/S9ddCY/wPBPD)

10. [De Masi MAN. 2009 Aplicações de isótopos estáveis de O, C e N em estudos de sazonalidade, mobilidade e dieta de populações pré-históricas no sul do Brasil. *Revista de Arqueologia* **22**, 55–76.](http://paperpile.com/b/S9ddCY/4mkIk)

11. [Wesolowski V, Neves WA. 2002 Economy, nutrition, and disease in prehistoric coastal Brazil: A case study for the State of Santa Catarina. In *The Backbone of History. Health and Nutrition in the Western Hemisphere* (eds RH Steckel, JC Rose), pp. 346–400. Cambridge: Cambridge University Press.](http://paperpile.com/b/S9ddCY/N3zq7)

12. [Buikstra J, Ubelaker D. 1994 *Standards for data collection from human skeletal remains*. Fayettville, Arkansas: Arkansas Archeological Survey Research Series.](http://paperpile.com/b/S9ddCY/LLwAJ)

13. [Wood JW *et al.* 1992 The osteological paradox: problems of inferring prehistoric health from skeletal samples [and Comments and Reply]. *Curr. Anthropol.* **33**, 343–370.](http://paperpile.com/b/S9ddCY/bbfbK)

14. [Pezo L, Eggers S. 2012 Caries through time: an anthropological overview. In *Contemporary Approach to Dental Caries* (ed M-Y Li), InTech.](http://paperpile.com/b/S9ddCY/qvgW8)

15. [Hillson S. 2001 Recording dental caries in archaeological human remains. *Int. J. Osteoarchaeol.* **11**, 249–289.](http://paperpile.com/b/S9ddCY/4nAh7)

16. [Pezo-Lanfranco L, Eggers S. 2010 The usefulness of caries frequency, depth, and location in determining cariogenicity and past subsistence: a test on early and later agriculturalists from the Peruvian coast. *Am. J. Phys. Anthropol.* **143**, 75–91.](http://paperpile.com/b/S9ddCY/9uFad)

17. [Pezo-Lanfranco L, Peralta A, Guillén S, Eggers S. 2017 Oral pathology patterns in late farmers of the Central Andes: A comparative perspective between coastal and highland populations. *Homo* **68**, 343–361.](http://paperpile.com/b/S9ddCY/9T3MP)

18. [Klein MI, Duarte S, Xiao J, Mitra S, Foster TH, Koo H. 2009 Structural and molecular basis of the role of starch and sucrose in Streptococcus mutans biofilm development. *Appl. Environ. Microbiol.* **75**, 837–841.](http://paperpile.com/b/S9ddCY/fFGIj)

19. [Brown TA, Nelson EE, Vogel SJ, Southon JR. 1988 Improved collagen extraction by modified longin method. *Radiocarbon* **30**, 171–177.](http://paperpile.com/b/S9ddCY/Iavo2)

20. [Craig OE, Biazzo M, Colonese AC, Di Giuseppe Z, Martinez-Labarga C, Lo Vetro D, Lelli R, Martini F, Rickards O. 2010 Stable isotope analysis of Late Upper Palaeolithic human and faunal remains from Grotta del Romito (Cosenza), Italy. *J. Archaeol. Sci.* **37**, 2504–2512.](http://paperpile.com/b/S9ddCY/H9QmK)

21. [Law IA, Hedges REM. 1989 A semi-automated bone pretreatment system and the pretreatment of older and contaminated samples. *Radiocarbon* **31**, 247–253.](http://paperpile.com/b/S9ddCY/bxyqj)

22. [Koch PL, Tuross N, Fogel ML. 1997 The Effects of Sample Treatment and Diagenesis on the Isotopic Integrity of Carbonate in Biogenic Hydroxylapatite. *J. Archaeol. Sci.* **24**, 417–429.](http://paperpile.com/b/S9ddCY/q2ci3)

23. [Ambrose SH. 1990 Preparation and characterization of bone and tooth collagen for isotopic analysis. *J. Archaeol. Sci.* , 431–451.](http://paperpile.com/b/S9ddCY/Q5j9K)

24. [Ambrose SH, Norr L. 1993 Experimental evidence for the relationship of the carbon isotope ratios of whole diet and dietary protein to those of bone collagen and carbonate. In *Prehistoric Human Bone - Archaeology at the Molecular Level*, pp. 1–37. Springer Verlag.](http://paperpile.com/b/S9ddCY/5IBHb)

25. [van Klinken GJ. 1999 Bone collagen quality indicators for palaeodietary and radiocarbon measurements. *J. Archaeol. Sci.* **26**, 687–695.](http://paperpile.com/b/S9ddCY/2duKC)

26. [DeNiro MJ. 1985 Postmortem preservation and alteration of in vivo bone collagen isotope ratios in relation to palaeodietary reconstruction. *Nature* **317**, 806–809.](http://paperpile.com/b/S9ddCY/DDbk0)

27. [Hammer Ø, Harper D, Ryan PD. 2001 PAST: Paleontological Statistics Software Package for education and data analysis. Palaeontologia Electronica 4.](http://paperpile.com/b/S9ddCY/HRXyL)

28. [Fernandes R, Millard AR, Brabec M, Nadeau M-J, Grootes P. 2014 Food reconstruction using isotopic transferred signals (FRUITS): a Bayesian model for diet reconstruction. *PLoS One* **9**, e87436.](http://paperpile.com/b/S9ddCY/9g9vV)

29. [Colonese AC *et al.* 2014 Long-term resilience of late holocene coastal subsistence system in Southeastern South america. *PLoS One* **9**, e93854.](http://paperpile.com/b/S9ddCY/F1nP4)

30. [Hardt FAS *et al.* 2013 Use of carbon and nitrogen stable isotopes to study the feeding ecology of small coastal cetacean populations in southern Brazil. *Biota Neotrop.* **13**, 90–98.](http://paperpile.com/b/S9ddCY/PBUhi)

31. [Hellevang H, Aagaard P. 2015 Constraints on natural global atmospheric CO2 fluxes from 1860 to 2010 using a simplified explicit forward model. *Sci. Rep.* **5**, 17352.](http://paperpile.com/b/S9ddCY/BIzpD)

32. [Fischer A, Olsen J, Richards M, Heinemeier J, Sveinbjörnsdóttir ÁE, Bennike P. 2007 Coast–inland mobility and diet in the Danish Mesolithic and Neolithic: evidence from stable isotope values of humans and dogs. *J. Archaeol. Sci.* **34**, 2125–2150.](http://paperpile.com/b/S9ddCY/W8VvG)

33. [Sholto-Douglas AD, Field JG, James AG, van der Merwe NJ. 1991 13C/12C and 15N/14N isotope ratios in the Southern Benguela Ecosystem: indicators of food web relationships among different size–classes of plankton and pelagic fish; differences between fish muscle and bone collagen tissues. *Mar Ecol Prog Ser* **78**, 23–31.](http://paperpile.com/b/S9ddCY/o5zeQ)

34. [Barros GV, Martinelli LA, Oliveira Novais TM, Ometto JPHB, Zuppi GM. 2010 Stable isotopes of bulk organic matter to trace carbon and nitrogen dynamics in an estuarine ecosystem in Babitonga Bay (Santa Catarina, Brazil). *Sci. Total Environ.* **408**, 2226–2232.](http://paperpile.com/b/S9ddCY/VfdO1)

35. [Angulo RJ, Giannini PCF, Suguio K, Pessenda LCR. 1999 Relative sea-level changes in the last 5500 years in southern Brazil (Laguna–Imbituba region, Santa Catarina State) based on vermetid 14C ages. *Mar. Geol.* **159**, 323–339.](http://paperpile.com/b/S9ddCY/WRg45)

36. [Angulo RJ, Lessa GC, Souza MC de. 2006 A critical review of mid- to late-Holocene sea-level fluctuations on the eastern Brazilian coastline. *Quat. Sci. Rev.* **25**, 486–506.](http://paperpile.com/b/S9ddCY/xvJf3)

37. [Behling H, Negrelle RRB. 2001 Tropical rain forest and climate dynamics of the atlantic lowland, southern Brazil, during the Late Quaternary. *Quat. Res.* **56**, 383–389.](http://paperpile.com/b/S9ddCY/FUI5L)

38. [Galetti M, Rodarte RR, Neves CL, Moreira M, Costa-Pereira R. 2016 Trophic niche differentiation in rodents and marsupials revealed by stable isotopes. *PLoS One* **11**, e0152494.](http://paperpile.com/b/S9ddCY/qqpJX)

39. [Boyadjian CHC, Eggers S, Reinhard KJ, Scheel-Ybert R. 2016 Dieta no sambaqui Jabuticabeira-II (SC): Consumo de plantas revelado por microvestígios provenientes de cálculo dentário. *Cadernos do LEPAARQ (UFPEL)* **13**, 131–161.](http://paperpile.com/b/S9ddCY/6c0XJ)

40. [Fernandes R, Grootes P, Nadeau M-J, Nehlich O. 2015 Quantitative diet reconstruction of a Neolithic population using a Bayesian mixing model (FRUITS): The case study of Ostorf (Germany). *Am. J. Phys. Anthropol.* (doi:](http://paperpile.com/b/S9ddCY/aIXX9)[10.1002/ajpa.22788](http://dx.doi.org/10.1002/ajpa.22788)[)](http://paperpile.com/b/S9ddCY/aIXX9)

41. [Fernandes R. 2016 A simple(R) model to predict the source of dietary carbon in individual consumers. *Archaeometry* **58**, 500–512.](http://paperpile.com/b/S9ddCY/44KM8)

42. [Colonese AC, Farrell T, Lucquin A, Firth D, Charlton S, Robson HK, Alexander M, Craig OE. 2015 Archaeological bone lipids as palaeodietary markers. *Rapid Commun. Mass Spectrom.* **29**, 611–618.](http://paperpile.com/b/S9ddCY/ZESBI)

43. [Fernandes R, Nadeau M-J, Grootes PM. 2012 Macronutrient-based model for dietary carbon routing in bone collagen and bioapatite. *Archaeol. Anthropol. Sci.* **4**, 291–301.](http://paperpile.com/b/S9ddCY/SxgDb)

44. [Jim S, Jones V, Ambrose SH, Evershed RP. 2006 Quantifying dietary macronutrient sources of carbon for bone collagen biosynthesis using natural abundance stable carbon isotope analysis. *Br. J. Nutr.* **95**, 1055–1062.](http://paperpile.com/b/S9ddCY/qxYyT)

45. [Webb EC, Lewis J, Shain A, Kastrisianaki-Guyton E, Honch NV, Stewart A, Miller B, Tarlton J, Evershed RP. 2017 The influence of varying proportions of terrestrial and marine dietary protein on the stable carbon-isotope compositions of pig tissues from a controlled feeding experiment. *STAR: Science & Technology of Archaeological Research* **3**, 36–52.](http://paperpile.com/b/S9ddCY/8Sie7)

46. [Bastos MQR, Lessa A, Rodrigues-Carvalho C, Tykot RH, Santos RV. 2014 Carbon and nitrogen isotope analysis: diet before and after the arrival of ceramic at Forte Marechal Luz Site. *Revista do Museu de Arqueologia e Etnologia* **0**, 137–151.](http://paperpile.com/b/S9ddCY/V5BKz)

47. [Bastos MQR, Santos RV, Tykot RH, Mendonça de Souza SMF, Rodrigues-Carvalho C, Lessa A. 2015 Isotopic evidences regarding migration at the archeological site of Praia da Tapera: New data to an old matter. *Journal of Archaeological Science: Reports* **4**, 588–595.](http://paperpile.com/b/S9ddCY/7lEQg)

48. [De Masi M. 2001 Pescadores coletores da costa sul do Brasil. *Pesquisas* **57**, 1–136.](http://paperpile.com/b/S9ddCY/CIzPE)

49. [Figuti L. 2009 Construindo o sambaqui: a ocupação e os processos de construção de sitio na bacia do Canal do Palmital, Santa Catarina.](http://paperpile.com/b/S9ddCY/zgFJd)
