# Supplementary material for: Middle Holocene plant cultivation on the Atlantic Forest coast of Brazil?
Source: R Soc Open Sci. 2018 Sep 5;5(9):180432. doi: 10.1098/rsos.180432 (PMC6170589; doi:10.1098/rsos.180432)
Supplement: Supplementary Material_Tables [file rsos180432supp2.docx]

**Supporting Information**

**Tables**

| S1 Table. Radiocarbon dates from human individuals from Rio Comprido (RC) and Morro do Ouro (MO). | | | | | | | | | |
| --- | --- | --- | --- | --- | --- | --- | --- | --- | --- |
| Phase | Sample | Level | Lab code | Material | ^14^C yr BP | ^14^C yr cal BP (1σ) | ^14^C yr cal BP (2σ) | Modelled ^14^C yr cal BP (2σ) | % marine C |
| RCII | RC4A | 0-1 m | Beta444032 | bone | 1140±30 | 1054 - 959 | 1061 - 936 | 925 - 699 | 59.5±6.0 |
| RCII | RC1A | 0-1 m | Beta448932 | bone | 3510±30 | 3826 - 3650 | 3835 - 3640 | 3608 - 3380 | 65.0±5.3 |
| RCII | RC3 | 0-1 m | Beta448933 | bone | 3820±30 | 4229 - 4090 | 4286 - 3990 | 4051 - 3712 | 66.7±5.1 |
| RCI | RC6 | 2-3 m | AA104771 | teeth | 4320±61 | 4960 - 4712 | 5039 - 4616 | 4800 - 4374 | 72.1±3.9 |
| RCI | RC68 | 5-6 m | Beta444033 | bone | 5090±30 | 5890 - 5740 | 5906 - 5664 | 5642 - 5438 | 70.4±4.4 |
| MO | MO13 | 0-1 m | AA104767 | teeth | 4425±39 | 5032 - 4869 | 5260 - 4846 | 4824 - 4527 | 73.2±3.6 |
| MO | MO59 | 2-3 m | AA104770 | bone | 3938±55 | 4415 - 4248 | 4514 - 4150 | 4510 - 4101 | 3.7±2.7 |
| MO | MO2 | 3-4 m | Beta444034 | bone | 4200±30 | 4820 - 4626 | 4830 - 4571 | 4530 - 4251 | 63.7±5.6 |
| MO | MO22 | 6-7 m | AA104768 | bone | 4086±42 | 4574 - 4435 | 4803 - 4417 | 4524 - 4244 | 60.9±5.8 |
| The ^14^C ages are reported as conventional and calibrated years BP using SHCal13 in OxCal v4.3 [(Hogg et al., 2013)](https://paperpile.com/c/YT7Bdn/h4AVE). Modelled ^14^C dates corrected for the reservoir effect according to the relative contribution of marine C to collagen. | | | | | | | | | |

| S2 Table: Demographic structure of Rio Comprido and Morro do Ouro | | | | | | | |
| --- | --- | --- | --- | --- | --- | --- | --- |
| Site/Phase | Age | | n | % | Sex | | |
|  |  |  |  |  | Male | Female | Und |
| Rio Comprido I  RC-I |  | Ch | 1 | 6.3 | 0 | 0 | 1 |
|  | Ad | | 2 | 12.5 | 0 | 1 | 1 |
|  | YA | | 7 | 43.8 | 4 | 2 | 1 |
|  | MA | | 5 | 31.3 | 3 | 2 | 0 |
|  | Adult | | 1 | 6.3 | 0 | 0 | 1 |
|  | **Total** | | **16** | **100.0** | **7** | **5** | **4** |
| Rio Comprido II  RC-II |  | Ch | 1 | 8.3 | 0 | 0 | 1 |
|  |  | Ad | 1 | 8.3 | 0 | 0 | 1 |
|  |  | YA | 2 | 16.7 | 2 | 0 | 0 |
|  |  | MA | 5 | 41.7 | 2 | 2 | 1 |
|  |  | Adult | 3 | 25.0 | 0 | 0 | 3 |
|  | **Total** | | **12** | **100.0** | **4** | **2** | **6** |
| Morro do Ouro  MO | Ch | | 1 | 2.3 | 0 | 0 | 1 |
|  | Ad | | 1 | 2.3 | 0 | 1 | 0 |
|  | YA | | 10 | 23.8 | 5 | 5 | 0 |
|  | MA | | 25 | 59.5 | 14 | 9 | 2 |
|  | Adult | | 5 | 11.9 | 0 | 1 | 4 |
|  | **Total** | | **42** | **100.0** | **19** | **16** | **7** |
| \| Pearson Chi-Square: *X*^2^=9.494; df=8; *p=*0.302 (based on 10000 replicates)  Jonckheere-Terpstra Test: Observed J-T Statistic =829.000; *p=*0.068 \| \| --- \|   Age class: Ch, Children (3-11 years); Ad, Adolescent (12-19 years); YA, young adult (20-29 years); MA, middle adult (30-49 years); OA (>50 years); Adult (no aged adult). | | | | | | | |

| S3 Table: Caries markers in Rio Comprido and Morro do Ouro | | | | | | | | | | | | | |
| --- | --- | --- | --- | --- | --- | --- | --- | --- | --- | --- | --- | --- | --- |
|  | Rio Comprido I (n=16) | | Rio Comprido II (n=12) | | Morro do Ouro (n=42) | | Total | Significance Chi square test (p<0.05) | | | | | |
|  |  |  |  |  |  |  |  | RCI vs. RC II | | RCI vs. MO | | RCII vs. MO | |
|  | n % | | n | % | n | % |  | *X^2^* | *p* | *X^2^* | *p* | *X^2^* | *p* |
| **Total alveoli** | **369** | **-** | **271** | **-** | **1187** | **-** | **1827** |  | |  | |  | |
| PMTL | 83 | 22.4 | 104 | 38.8 | 241 | 20.3 | 428 | 20.6263 | **0.0000** | 0.6880 | 0.4068 | 41.9220 | **0.0000** |
| AMTL | 23 | 6.2 | 03 | 1.1 | 29 | 2.4 | 55 | 10.5342 | **0.0012** | 12.5169 | **0.0004** | 1.8349 | 0.1756 |
| Total teeth | 263 | - | 164 | - | 917 | - | 1344 |  |  |  |  |  |  |
| Carious teeth | 20 | 7.6 | 20 | 12.3 | 121 | 13.2 | 161 | 1.0240 | 0.3115 | 7.7840 | **0.0052** | 2.0000 | 0.1573 |
| Caries depth | | | | | | | | | | | | | |
| 1. Enamel | 16 | 80.0 | 11 | 55.0 | 73 | 60.3 | 100 | 2.8490 | 0.0914 | 2.8525 | 0.0912 | 0.2025 | 0.6527 |
| 1. Dentin | 3 | 15.0 | 4 | 20.0 | 28 | 23.1 | 35 | 0.1732 | 0.6773 | 0.6631 | 0.4155 | 0.0965 | 0.7561 |
| 1. Pulp | 1 | 5.0 | 5 | 25.0 | 16 | 13.2 | 22 | 3.1373 | 0.0765 | 1.8780 | 0.1706 | 1.0946 | 0.2955 |
| 1. Gross-gross caries | 0 | 0.0 | 0 | 0.0 | 4 | 3.3 | 4 | 0.0000 | 1.0000 | 0.6805 | 0.4094 | 0.6805 | 0.4094 |
| Caries type | | | | | | | | | | | | | |
| 1. Occlusal | 14 | 70.0 | 13 | 65.0 | 65 | 53.7 | 92 | 0.1140 | 0.7356 | 1.8470 | 0.1741 | 0.8837 | 0.3472 |
| 1. Pit caries | 2 | 10.0 | 1 | 5.0 | 4 | 3.3 | 7 | 0.3604 | 0.5483 | 1.8878 | 0.1695 | 0.1440 | 0.7043 |
| 1. Smooth surface B/L | 0 | 0.0 | 1 | 5.0 | 3 | 2.5 | 4 | 1.0256 | 0.3112 | 0.5066 | 0.4766 | 0.3956 | 0.5294 |
| 1. Approximal | 0 | 0.0 | 0 | 0.0 | 1 | 0.8 | 1 | 0.0000 | 1.0000 | 0.1665 | 0.6833 | 0.1665 | 0.6833 |
| 1. Smooth surface M/D | 0 | 0.0 | 0 | 0.0 | 0 | 0.00 | 0 | 0.0000 | 1.0000 | 0.0000 | 1.0000 | 0.0000 | 1.0000 |
| 1. Cervical and/or CEJ | 3 | 15.0 | 0 | 0.0 | 35 | 28.9 | 39 | 1.1111 | 0.2918 | 1.6906 | 0.1935 | 5.1674 | **0.0230** |
| 1. Tooth wear, chipping and caries | 0 | 0.0 | 0 | 0.0 | 0 | 0.0 | 0 | 0.0000 | 1.0000 | 0.0000 | 1.0000 | 0.0000 | 1.0000 |
| 1. Tooth wear, pulp exposure and caries | 1 | 5.0 | 5 | 25.0 | 9 | 7.4 | 14 | 3.1370 | 0.0765 | 0.1548 | 0.6940 | 5.9190 | **0.0149** |
| 1. Gross-gross caries | 0 | 0.0 | 0 | 0.0 | 4 | 3.3 | 4 | 0.0000 | 1.0000 | 0.6805 | 0.4094 | 0.6805 | 0.4094 |
| Extra-occlusal (categories 3-6) | 3 | 15.0 | 1 | 5.00 | 39 | 32.23 | 44 | 0.1110 | 0.2918 | 2.4366 | 0.1185 | 6.2630 | **0.0123** |

| S4 Table: Dental calculus, alveolar resorption and dental wear indexes in  Rio Comprido and Morro do Ouro | | | | | | | | | |
| --- | --- | --- | --- | --- | --- | --- | --- | --- | --- |
| Population | Dental calculus | | | Alveolar resorption | | | Dental wear | | |
|  | n | Mean | SD | n | Mean | SD | n | Mean | SD |
| Rio Comprido I | 9 | 2.1 | 0.8 | 11 | 1.3 | 0.8 | 16 | 4.2 | 1.5 |
| Rio Comprido II | 6 | 2.5 | 0.8 | 7 | 1.5 | 1.0 | 12 | 5.2 | 1.4 |
| Morro do Ouro | 36 | 2.2 | 0.7 | 39 | 1.2 | 0.5 | 42 | 4.6 | 1.3 |
| Kruskal-Wallis Test | *X*^2^=2.005; df=2; *p*=0.367 | | | *X*^2^=1.656; df=2; *p*=0.437 | | | *X*^2^=3.614; df=2; *p*=0.164 | | |

| S5 Table: Prevalence of oral pathologies per population* | | | | | | | | | | | | |
| --- | --- | --- | --- | --- | --- | --- | --- | --- | --- | --- | --- | --- |
|  | Rio Comprido I  (N=16) | | Rio Comprido II  (N=12) | | Morro do Ouro  (N=42) | | Chi square test (p<0.05) | | | | | |
|  |  |  |  |  |  |  | RCI-RCII | | RCI-MO | | RCII-MO | |
|  |  |  |  |  |  |  | *X^2^* | *p* | *X^2^* | *p* | *X^2^* | *p* |
|  | n | % | n | % | n | % |  |  |  |  |  |  |
| Caries | 08(16) | 50.0 | 07(12) | 58.3 | 31(42) | 70.4 | 0.1915 | 0.6617 | 2.9818 | 0.0842 | 1.0721 | 0.3005 |
| AMTL | 04(16) | 25.0 | 02(12) | 16.6 | 08(42) | 25.9 | 0.2828 | 0.5649 | 0.2502 | 0.6170 | 0.0351 | 0.8515 |
| Periodontal disease** | 03(10) | 30.0 | 03(06) | 50.0 | 10(40) | 25.9 | 0.6400 | 0.4237 | 0.1040 | 0.7471 | 1.6084 | 0.2047 |
| Periapical lesions | 02(16) | 12.5 | 04(12) | 33.3 | 17(42) | 40.7 | 1.7677 | 0.1837 | 4.1168 | **0.0425** | 0.2004 | 0.6544 |
| *Individuals affected by pathological condition among the evaluated individuals (condition ≥1). **In brackets the number of individuals with observable parameters. Chi-square Test was applied for inter-group comparisons. | | | | | | | | | | | | |

| S6 Table: Quality indicators and stable isotope values of bulk dentine collagen and enamel carbonate from RCI, RCII and MO. The estimated relative caloric contribution of food sources are also reported (P, plant; F, fish; TM, terrestrial mammals). Model estimates using bulk dentine collagen (δ^13^C_col_ and δ^15^N_col_) and enamel carbonate (δ^13^C_ap_), and bulk dentine collagen only (*) | | | | | | | | | | | | | | | | |  |
| --- | --- | --- | --- | --- | --- | --- | --- | --- | --- | --- | --- | --- | --- | --- | --- | --- | --- |
| Site/Phase | Individual | Age at death | Sex | Sample | δ^13^C_ap_ | δ^13^C_col_ | δ^15^N_col_ | C% | N% | C/N | P% | F% | TM% | *P% | *F% | *TM% | |
| RCI | RC 6 | MA | M | PM2(4,5) | -9.0 | -13.1 | +15.2 | 35.3 | 11.7 | 3.2 | 33±5 | 58±3 | 9±8 | 36±12 | 50±6 | 13±12 |  |
| RCI | RC 37A | MA | F | PM2(4,5) | -8.4 | -13.9 | +14.5 | 25.2 | 7.3 | 3.4 | 32±7 | 58±4 | 11±10 | 41±12 | 46±6 | 13±13 |  |
| RCI | RC 46 | YA | M | M3(4,8) | -8.5 | -13.8 | +15.7 | 28.3 | 8.3 | 3.6 | 31±7 | 57±4 | 12±10 | 36±13 | 47±6 | 17±14 |  |
| RCI | RC 68 | MA | M | PM2(1,5) | -7.3 | -13.4 | +14.8 | 28.6 | 8.5 | 3.5 | 34±4 | 60±3 | 6±6 | 39±12 | 48±6 | 13±12 |  |
| MO | MO 13 | MA | F | M3(2,8) | -8.0 | -13.4 | +15.4 | 36.5 | 11.3 | 3.3 | 33±5 | 59±3 | 8±7 | 36±12 | 49±6 | 15±13 |  |
| MO | MO 22 | YA | M | M3(3,8) | -10.2 | -14.6 | +13.4 | 21.3 | 6.5 | 3.4 | 28±12 | 51±5 | 22±16 | 51±11 | 39±6 | 10±10 |  |
| MO | MO 28 | Adult | F | PM1(1,4) | -9.7 | -13.9 | +15.2 | 34.0 | 9.8 | 3.4 | 29±9 | 54±5 | 17±12 | 38±13 | 46±6 | 16±14 |  |
| MO | MO 44 | MA | F | PM2(1,5) | -10.6 | -15.7 | +13.0 | 19.6 | 5.7 | 3.5 | 21±15 | 44±6 | 36±19 | 59±10 | 32±6 | 9±10 |  |
| MO | MO 59 | YA | F | PM2(4,5) | -12.3 | -21.3 | +10.0 | 22.6 | 6.3 | 3.6 | 92±3 | 5±2 | 3±3 | 86±7 | 5±6 | 9±8 |  |
| MO | MO 60 | MA | M | M2(4,7) | -10.5 | -15.5 | +13.2 | 34.2 | 10.4 | 3.3 | 21±15 | 45±6 | 34±19 | 57±12 | 34±6 | 10±11 |  |
| MO | MO 72 | Adult | Und | M3(3,8) | -7.4 | -13.7 | +14.9 | 18.4 | 4.8 | 3.6 | 33±5 | 60±3 | 8±7 | 39±12 | 47±6 | 14±12 |  |
| MO | MO 77 | MA | M | PM2(1,5) | -8.9 | -12.9 | +14.1 | 32.0 | 10.6 | 3.2 | 33±5 | 59±3 | 8±7 | 39±10 | 51±6 | 10±10 |  |
| MO | MO 84A | YA | F | PM1(1,4) | -9.9 | -13.8 | +14.7 | 33.5 | 9.9 | 3.3 | 30±9 | 54±5 | 15±11 | 40±12 | 46±6 | 14±13 |  |
| Teeth and age of eruption: PM1=1st permanent premolar (2-11 years); PM2=2nd permanent premolar (3-12 years); M2=2nd permanent molar (4-14 years); M3=3rd permanent molar (10-20 years). In brackets the number of tooth in FDA notation. Age class: Ch, Children (3-11 years); Ad, Adolescent (12-19 years); YA, young adult (20-29 years); MA, middle adult (30-49 years); OA (>50 years); Adult (no aged adult). | | | | | | | | | | | | | | | | |  |

| S7 Table: Quality indicators and stable isotope values of bone bulk collagen from RCI, RCII and MO. The estimated relative caloric contribution of food sources are also reported (P, plant; F, fish; TM, terrestrial mammals). | | | | | | | | | | | | | |
| --- | --- | --- | --- | --- | --- | --- | --- | --- | --- | --- | --- | --- | --- |
| Site/phase | Individual | Age at death | Sex | Sample | δ^13^C_col_ | δ^15^N_col_ | C% | N% | C/N | P% | F% | TM% |  |
| RCI | RC 42B | Ch (~8y) | Und | Rib | -15.2 | +12.4 | 35.1 | 12.3 | 3.3 | 59±10 | 34±6 | 7±8 |  |
| RCI | RC 52 | Ad | F | Cranial | -14.1 | +11.7 | 16.2 | 5.5 | 3.4 | 54±9 | 41±7 | 5±6 |  |
| RCI | RC 66 | YA | M | Rib | -12.5 | +13.4 | 37.8 | 13.2 | 3.3 | 40±9 | 53±6 | 8±8 |  |
| RCI | RC 67 A | YA | F | Rib | -13.0 | +12.8 | 26.0 | 9.1 | 3.3 | 43±9 | 50±6 | 8±8 |  |
| RCI | RC 67 D | YA | F | Cranial | -12.2 | +14.8 | 40.5 | 14.5 | 3.3 | 36±9 | 55±6 | 10±9 |  |
| RCI | RC 68 | MA | M | Cranial | -11.4 | +15.0 | 38.9 | 13.8 | 3.3 | 35±7 | 57±5 | 8±7 |  |
| RCII | RC 1A | MA | F | Rib | -12.6 | +13.6 | 26.9 | 9.1 | 3.5 | 40±9 | 52±6 | 8±8 |  |
| RCII | RC 2A | Ch (~6y) | Und | Rib | -13.4 | +12.2 | 21.1 | 7.0 | 3.5 | 47±9 | 46±7 | 7±7 |  |
| RCII | RC 3 | Adult | M | Rib | -12.2 | +12.9 | 32.7 | 11.4 | 3.3 | 40±8 | 53±6 | 7±7 |  |
| RCII | RC 4A | MA | M | Rib | -13.9 | +16.3 | 41.0 | 14.6 | 3.3 | 34±13 | 47±6 | 19±14 |  |
| RCII | RC 22 | Adult | Und | Rib | -13.9 | +12.4 | 33.1 | 10.9 | 3.5 | 50±10 | 43±7 | 7±8 |  |
| RCII | RC 30A | YA | M | Rib | -13.4 | +12.9 | 17.5 | 5.8 | 3.6 | 45±10 | 47±6 | 8±8 |  |
| RCII | RC 44 | MA | F | Rib | -13.4 | +14.5 | 35.5 | 12.7 | 3.3 | 39±11 | 48±6 | 12±11 |  |
| MO | MO 2 | MA | F? | Rib | -12.9 | +13.3 | 35.7 | 12.5 | 3.3 | 41±9 | 51±6 | 8±8 |  |
| MO | MO 14 | MA | F | Rib | -13.3 | +13.3 | 29.2 | 9.9 | 3.5 | 43±10 | 48±6 | 9±9 |  |
| MO | MO 22 | YA | M | Rib | -13.4 | +13.8 | 37.6 | 13.1 | 3.4 | 42±11 | 48±6 | 10±10 |  |
| MO | MO 23 | MA | M | Rib | -14.1 | +13.2 | 38.3 | 13.3 | 3.4 | 49±11 | 43±6 | 9±9 |  |
| MO | MO 28 | Adult | F | Rib | -12.4 | +13.6 | 30.1 | 10.4 | 3.4 | 39±9 | 53±6 | 8±8 |  |
| MO | MO 29 | MA | M | Rib | -17.1 | +12.5 | 32.0 | 10.9 | 3.4 | 69±10 | 24±5 | 8±10 |  |
| MO | MO 31 | Adult | Und | Rib | -12.9 | +13.4 | 31.2 | 10.6 | 3.4 | 41±10 | 51±6 | 9±8 |  |
| MO | MO 38 | YA | M | Rib | -13.2 | +14.0 | 36.2 | 12.7 | 3.3 | 41±10 | 49±6 | 10±10 |  |
| MO | MO 47 | MA | M | Rib | -12.0 | +14.1 | 29.2 | 10.1 | 3.4 | 37±8 | 55±6 | 8±8 |  |
| MO | MO 52 | MA | M | Rib | -13.0 | +13.0 | 30.5 | 10.7 | 3.3 | 43±10 | 49±7 | 8±8 |  |
| MO | MO 56 | YA | F | Cranial | -13.8 | +12.2 | 25.1 | 8.6 | 3.4 | 50±10 | 44±6 | 6±7 |  |
| MO | MO 57 | OA | M? | Rib | -12.3 | +13.2 | 19.3 | 6.5 | 3.5 | 39±9 | 53±6 | 8±8 |  |
| MO | MO 59 | YA | F | Rib | -20.6 | +7.0 | 16.7 | 5.7 | 3.5 | 95±3 | 2±2 | 3±3 |  |
| MO | MO 60 | MA | M | Rib | -14.3 | +12.0 | 28.4 | 9.8 | 3.4 | 55±9 | 39±6 | 6±6 |  |
| MO | MO 82A | MA | F | Rib | -13.4 | +12.9 | 28.2 | 9.8 | 3.4 | 45±10 | 47±6 | 8±8 |  |
| MO | MO 84B | Ch (~4y) | Und | Rib | -13.8 | +14.5 | 37.2 | 13.1 | 3.3 | 41±12 | 46±6 | 12±12 |  |
| Age class: Ch, Children (3-11 years); Ad, Adolescent (12-19 years); YA, young adult (20-29 years); MA, middle adult (30-49 years); OA (>50 years); Adult (no aged adult). | | | | | | | | | | | | | |

| S8Table: Isotope values of food source fractions derived from bulk collagen and bulk plant isotope values using offsets reported in the text. | | | | |
| --- | --- | --- | --- | --- |
| Food sources | Fraction | δ^13^C_col_ | δ^15^N_col_ | δ^13^C_ap_ |
| Plant | Bulk | - | - | -31.2 ± 1 |
| Plant | Protein | -31.2 ± 1 | +1.1 ± 1 | - |
| Plant | Carbohydrates | -28.7 ± 1 | - | - |
| T. mammals | Bulk | - | - | -28.2 ± 1 |
| T. mammals | Protein | -24.0 ± 1 | +10.4 ± 1 | - |
| T. mammals | Lipids | -30.0 ± 1 | - | - |
| Fish | Bulk | - | - | -14.5 ± 1 |
| Fish | Protein | -12.9 ± 1 | +14.8 ± 1 | - |
| Fish | Lipids | -18.9 ± 1 | - | - |
